# Cross-sectional and longitudinal analyses of outdoor air pollution exposure and cognitive function in UK Biobank

**DOI:** 10.1038/s41598-018-30568-6

**Published:** 2018-08-14

**Authors:** Breda Cullen, Danielle Newby, Duncan Lee, Donald M. Lyall, Alejo J. Nevado-Holgado, Jonathan J. Evans, Jill P. Pell, Simon Lovestone, Jonathan Cavanagh

**Affiliations:** 1 Institute of Health and Wellbeing, University of Glasgow, Glasgow, United Kingdom; 2 Department of Psychiatry, University of Oxford, Oxford, United Kingdom; 3 School of Mathematics and Statistics, University of Glasgow, Glasgow, United Kingdom

## Abstract

Observational studies have shown consistently increased likelihood of dementia or mild cognitive impairment diagnoses in people with higher air pollution exposure history, but evidence has been less consistent for associations with cognitive test performance. We estimated the association between baseline neighbourhood-level exposure to airborne pollutants (particulate matter and nitrogen oxides) and (1) cognitive test performance at baseline and (2) cognitive score change between baseline and 2.8-year follow-up, in 86,759 middle- to older-aged adults from the UK Biobank general population cohort. Unadjusted regression analyses indicated small but consistent negative associations between air pollutant exposure and baseline cognitive performance. Following adjustment for a range of key confounders, associations were inconsistent in direction and of very small magnitude. The largest of these indicated that 1 interquartile range higher air pollutant exposure was associated on average with 0.35% slower reaction time (95% CI: 0.13, 0.57), a 2.92% higher error rate on a visuospatial memory test (95% CI: 1.24, 4.62), and numeric memory scores that were 0.58 points lower (95% CI: −0.96, −0.19). Follow-up analyses of cognitive change scores did not show evidence of associations. The findings indicate that in this sample, which is five-fold larger than any previous cross-sectional study, the association between air pollution exposure and cognitive performance was weak. Ongoing follow-up of the UK Biobank cohort will allow investigation of longer-term associations into old age, including longitudinal tracking of cognitive performance and incident dementia outcomes.

## Introduction

Outdoor air pollutants such as particulate matter (PM), nitrogen oxides (NO_x_), carbon monoxide (CO) and ground-level ozone (O_3_) are associated with acute and chronic illnesses including cancer, asthma, stroke, heart disease and diabetes^1^. Possible links with cognitive decline have received research attention over the past decade, summarised in five recent systematic reviews^2–6^. Although confounding background factors such as deprivation and education may partially account for observed associations with cognitive outcomes, it has been postulated that exposure to air pollution has a causal influence via harmful effects on the brain. There is evidence from animal and human studies that air pollutants are linked to inflammatory processes and morphological changes in the brain, such as upregulation of pro-inflammatory cytokines (including interleukin-1 alpha and beta, interleukin-6 and tumour necrosis factor alpha), activation of microglia and reactive oxygen species, alpha-synuclein deposition, and decreases in dendritic spine density and branching in the hippocampus^7–11^. These may result from infiltration of pollutants such as ultrafine particulate matter into brain tissue, via the olfactory nerve or the blood-brain barrier^11,12^, with possible contributions through pulmonary inflammation and downstream effects on systemic circulation^13^. Cardiovascular and cerebrovascular morbidity and mortality are elevated in association with air pollution exposure^14–16^, and neuroimaging studies have shown associations with atrophy, infarcts, white matter hyperintensities and altered cerebrovascular haemodynamics^17,18^.

Associations of generally small effect size have been reported in cross-sectional studies of air pollution exposure and cognitive test performance^19–26^, although others have reported primarily null results^27,28^. Inconsistent small or null effects have also been shown in studies of cognitive score change over intervals of two to five years^29–31^. A smaller number of studies have examined associations with clinical cognitive disorders. In cross-sectional and case-control studies, air pollutant exposure has been linked with greater odds of global and amnestic mild cognitive impairment (MCI)^32^ and Alzheimer’s disease or vascular dementia^33^. Cohort studies investigating dementia incidence over follow-up periods of up to 15 years have reported greater hazard ratios associated with air pollutant exposure^34–36^ and proximity to major roads^37^. Heterogeneity of study populations, measures and statistical adjustment methods means that none of the five systematic reviews published to date has included a meta-analysis of effect size estimates. Considerable uncertainty therefore remains regarding the likely magnitude of any association.

Large study samples are needed for the reliable detection of associations of small effect size. Cross-sectional studies investigating the relationship between air pollution exposure and performance on cognitive tests have used samples ranging from *n* = 399^22^ to *n* = 15,973^26^, and longitudinal studies of cognitive score change have ranged from *n* = 2,867^30^ to *n* = 20,150^29^. The inconsistent findings and imprecision of the effect size estimates from these studies may indicate the need for samples which are larger still. Previous studies have also varied considerably in the air pollutant exposures that have been measured—most frequently particulate matter, with gas pollutants studied less often—and in the outcomes investigated, encompassing a range of different cognitive assessments or dementia diagnoses. Furthermore, the type and number of possible confounding variables adjusted in statistical analyses have varied widely: most studies have adjusted for sociodemographic factors (including age, gender, ethnicity, education and socioeconomic status), and some have taken account of lifestyle factors such as smoking and physical activity. No study to date has examined the influence of time spent outdoors, despite the fact that the pollution measures predominantly capture neighbourhood air quality rather than individual-level exposure; it might be expected that the magnitude of the relationship between neighbourhood pollution measures and cognitive outcomes would be greater among those who spend more time outdoors.

The UK Biobank resource^38^ offers an opportunity to address some of these limitations. More than half a million adults in middle to early old age were included in the UK Biobank cohort at baseline. Importantly, although the air pollution measures were taken at the neighbourhood level, participants also provided individual information regarding length of time typically spent outdoors. Moreover, a subset of the cohort returned for repeat cognitive assessment between two and seven years post-baseline, thus permitting both cross-sectional and longitudinal analyses. This is the largest study to date of air pollution and cognitive test performance, with standardised measurement of multiple pollutant exposures, cognitive outcomes and potential confounders, in a single general population cohort.

The aims of this study were to estimate the association between baseline neighbourhood-level exposure to airborne pollutants and (1) cognitive test performance at baseline and (2) cognitive score change between baseline and follow-up, taking account of confounding factors and the potential moderating influence of time spent outdoors.

## Results

### Characteristics of the study population

Of the full cohort (*n* = 502,623), 88,277 had their baseline assessment on or after 01 January 2010, of whom 86,759 (98.3%) had data on at least one air pollution measure and cognitive test. Of these, 2,913 (3.4%) attended the follow-up visit, which took place a mean of 2.8 years (standard deviation [SD] 0.2) after baseline, and had repeat data on at least one cognitive test. Table 1 summarises the baseline characteristics of the participants included in the cross-sectional and follow-up analyses. Descriptive information indicated that the sub-group of participants included in the follow-up sample differed in their baseline characteristics from the cross-sectional sample: the follow-up sub-group was somewhat older at baseline, resided in less deprived and less polluted neighbourhoods, scored better on the baseline cognitive tests, and had relatively higher proportions of men, white participants, never-smokers, participants with a degree, and non-urban dwellers. The follow-up sample also had lower proportions of missing data on most measures, although missingness was generally low across all baseline measures with the exception of physical activity (7.5% missing) and time spent outdoors (6.8% missing).

**Table 1:** Baseline characteristics of UK Biobank participants included in cross-sectional and follow-up analyses.

	Cross-sectional analysis sample	Follow-up analysis sample
*n*	86,759	2,913
**Age (years)** ^**a**^		
Mean ± SD	56.86 ± 8.12	58.30 ± 7.06
**Gender** ^**a**^		
*n* (%) female	47,205 (54.41)	1,498 (51.42)
**Ethnic group**		
*n* (%) missing	417 (0.48)	3 (0.10)
White, *n* (%)^b^	78,372 (90.77)	2,875 (98.80)
Asian/Asian British	2,790 (3.23)	5 (0.17)
Black/black British	2,789 (3.23)	8 (0.27)
Chinese	380 (0.44)	4 (0.14)
Mixed and Other	2,011 (2.33)	18 (0.62)
**Townsend deprivation score**		
*n* (%) missing	111 (0.13)	3 (0.10)
Median (Q1, Q3)	−1.68 (−3.35, 0.96)	−3.13 (−4.10, −1.66)
**Educational attainment**		
*n* (%) missing	985 (1.14)	4 (0.14)
Has a degree, *n* (%)^b^	29,886 (34.84)	1,372 (47.16)
**Smoking status**		
*n* (%) missing	371 (0.43)	7 (0.24)
Ever smoker, *n* (%)^b^	38,344 (44.39)	1,113 (38.30)
**Physical activity (MET h/week)**		
*n* (%) missing	6,513 (7.51)	164 (5.63)
Median (Q1, Q3)	27.93 (12.78, 57.53)	27.43 (12.60, 54.38)
**Time spent outdoors (h/day)**		
*n* (%) missing	5,857 (6.75)	130 (4.46)
Median (Q1, Q3)	2.5 (1.5, 3.5)	2.5 (1.5, 3.5)
**Neurological condition (self-reported)** ^**c**^		
Yes, *n* (%)	3,642 (4.20)	123 (4.22)
**Inverse distance from nearest major road (1/m)** ^**a**^		
Median (Q1, Q3)	0.003 (0.001, 0.006)	0.002 (0.001, 0.006)
**Traffic intensity on nearest major road (2008) (vehicles/24 h)** ^**a**^		
Median (Q1, Q3)	18561 (14343, 25211)	14674 (9918, 20648)
**Noise pollution (2009) (average dB/24 h)** ^**a**^		
Median (Q1, Q3)	54.82 (53.57, 56.81)	55.06 (53.37, 57.31)
**Population density category (2001)** ^**d**^		
*n* (%) missing	876 (1.01)	33 (1.13)
England/Wales urban less sparse, *n* (%)^b^	77,502 (90.24)	2,042 (70.90)
England/Wales town & fringe less sparse	4,245 (4.94)	325 (11.28)
England/Wales village less sparse	2,852 (3.32)	359 (12.47)
England/Wales hamlet & isolated less sparse	1,279 (1.49)	154 (5.35)
England/Wales other	5 (0.01)	0 (0)
**PM**_**10**_ **(2007) (μg/m** ^**3**^ **)** ^**e**^		
*n* (%) missing	128 (0.15)	0 (0)
Median (Q1, Q3)	22.19 (20.95, 25.07)	20.81 (19.30, 22.29)
**PM**_**2**. **5 to 10**_ **(2010) (μg/m** ^**3**^ **)** ^**e**^		
*n* (%) missing	33 (0.04)	1 (0.03)
Median (Q1, Q3)	6.20 (5.91, 6.68)	6.01 (5.80, 6.32)
**PM**_**2**. **5**_ **(2010) (μg/m** ^**3**^ **)** ^**e**^		
*n* (%) missing	33 (0.04)	1 (0.03)
Median (Q1, Q3)	9.86 (9.32, 10.40)	9.55 (8.86, 10.16)
**NO**_**2**_ **(2005) (μg/m** ^**3**^ **)** ^**a**, **e**^		
Median (Q1, Q3)	33.20 (26.20, 39.91)	25.35 (20.54, 30.04)
**NO**_**x**_ **(2010) (μg/m** ^**3**^ **)** ^**a**, **e**^		
Median (Q1, Q3)	43.29 (35.97, 50.64)	36.47 (28.20, 43.66)
**Reasoning score(range 0–13)**		
*n* (%) missing	3,395 (3.91)	22 (0.76)
Mean ± SD	5.99 ± 2.17	6.86 ± 2.01
**Reaction time (ms)**		
*n* (%) missing	980 (1.13)	11 (0.38)
Median (Q1, Q3)	546 (484, 624)	535 (483, 609)
**Numeric memory score (range 2–12)**		
*n* (%) missing^f^	34 (2.28)	
Mean ± SD	6.73 ± 1.27	^g^
**Pairs matching errors** ^**a**^		
Median (Q1, Q3)	3 (2, 5)	3 (2, 5)
**Prospective memory**		
*n* (%) missing	433 (0.50)	3 (0.10)
Correct on first attempt, *n* (%)	66,503 (77.04)	2,534 (87.08)

MET, metabolic equivalent of task; NO_2_, nitrogen dioxide; NO_x_, nitrogen oxides; PM, particulate matter; Q, quartile; SD, standard deviation.

^a^No missing data.

^b^Missing excluded from denominator.

^c^Not possible to distinguish between missing data and self-report of no condition, therefore both classified as No.

^d^Output areas classified by settlement type (urban [population > 10,000]; town & fringe; village; hamlet & isolated dwelling) and density (sparse; less sparse).

^e^EU data linked centrally by UK Biobank.

^f^Available *n* for this test was 1,458. Missing data refers only to the period when this test was included in the battery.

^g^Results not reported in the follow-up analysis sample because this test was not included in the protocol in the follow-up center, at either baseline or follow-up visits.

The median change score on the reasoning test was 0 (interquartile range [IQR] = 2; *n* = 2,878), and was also 0 on the pairs matching test (IQR = 4; *n* = 2,913). The median change on the reaction time test was −5 ms (indicating faster performance at follow-up; IQR = 109; *n* = 2,896). Of *n* = 2,910 with follow-up data on the prospective memory test, 121 (4.2%) showed decline.

### Association between air pollution exposure and baseline cognitive function

Table 2 shows the results of the separate regression models for each air pollution exposure and each cognitive test at baseline. In the unadjusted models, associations were evident between all five air pollutant measures and four of the five cognitive scores, with higher levels of pollutants being associated with worse cognitive performance. Point estimates for the numeric memory test followed the same pattern as for the other cognitive tests, but the sample sizes on this test (≤1,458) were considerably smaller than for the other tests (≥83,238) and the confidence intervals included the null.

**Table 2:** Results of regression models for cognitive performance in UK Biobank participants at baseline (2010), using centrally-linked pollutant data.

Exposure (year)	Cognitive score	Unadjusted						Adjusted^a^					
		*n*	Estimate	(95% CI)	*p* (uncorr)	*p* (FDR)^b^	+/−	*n*	Estimate	(95% CI)	*p* (uncorr)	*p* (FDR)^b^	+/−
PM_10_ (2007)	Reasoning^c^	83,238	−0.0139	(−0.0187, −0.0091)	1.558e-08	2.050e-08	−	72,004	0.0111	(0.0054, 0.0169)	0.0001	0.0025	+
	Reaction time^d^	85,651	1.0019	(1.0015, 1.0023)	3.245e-18	5.795e-18	−	73,581	0.9995^e^	(0.9986, 1.0004)	0.2799	0.3888	+
	Numeric memory^f^	1,456	−0.0301	(−0.0762, 0.0160)	0.2003	0.2177	−	1,258	−0.0285	(−0.0882, 0.0312)	0.3492	0.4595	−
	Pairs matching^g^	83,806	1.0083	(1.0065, 1.0101)	2.295e-20	4.781e-20	−	72,338	1.0019	(0.9997, 1.0042)	0.0907	0.1512	−
	Prospective memory^h^	86,198	0.9649	(0.9601, 0.9696)	2.986e-46	1.493e-45	−	73,974	1.0067	(0.9994, 1.0141)	0.0738	0.1419	+
PM_2.5 to 10_ (2010)	Reasoning^c^	83,331	−0.0915	(−0.1092, −0.0737)	4.334e-24	1.083e-23	−	72,079	−0.0103	(−0.0334, 0.0128)	0.3830	0.4788	−
	Reaction time^d^	85,746	1.0049	(1.0033, 1.0065)	1.996e-09	2.772e-09	−	73,656	1.0019	(0.9997, 1.0042)	0.0887	0.1512	−
	Numeric memory^f^	1,458	−0.0533	(−0.1237, 0.0171)	0.1376	0.1564	−	1,260	−0.2051^i^	(−0.3706, −0.0395)	0.0152	0.0556	−
	Pairs matching^g^	83,901	1.0159	(1.0094, 1.0225)	1.644e-06	2.055e-06	−	72,413	0.9966	(0.9874, 1.0059)	0.4727	0.5372	+
	Prospective memory^h^	86,293	0.9217	(0.9053, 0.9384)	4.855e-19	9.337e-19	−	74,049	0.9963	(0.9674, 1.0261)	0.8061	0.8397	−
PM_2.5_ (2010)	Reasoning^c^	83,331	−0.1196	(−0.1361, −0.1031)	1.212e-45	5.050e-45	−	72,079	−0.0141	(−0.0347, 0.0065)	0.1802	0.2816	−
	Reaction time^d^	85,746	1.0079	(1.0064, 1.0093)	4.543e-26	1.420e-25	−	73,656	1.0032	(1.0012, 1.0053)	0.0019	0.0158	−
	Numeric memory^f^	1,458	−0.0382	(−0.1018, 0.0255)	0.2396	0.2496	−	1,260	−0.0876	(−0.1797, 0.0045)	0.0624	0.1300	−
	Pairs matching^g^	83,901	1.0211	(1.0149, 1.0274)	2.653e-11	3.901e-11	−	72,413	1.0022	(0.9938, 1.0106)	0.6133	0.6666	−
	Prospective memory^h^	86,293	0.8741	(0.8592, 0.8893)	1.032e-52	8.600e-52	−	74,049	0.9890	(0.9629, 1.0157)	0.4156	0.4948	−
NO_2_ (2005)	Reasoning^c^	83,364	−0.0056	(−0.0070, −0.0041)	1.388e-14	2.169e-14	−	72,106	0.0032	(0.0013, 0.0050)	0.0007	0.0088	+
	Reaction time^d^	85,779	1.0007	(1.0006, 1.0008)	1.833e-29	6.546e-29	−	73,683	0.9999^j^	(0.9997, 1.0003)	0.9088	0.9088	+
	Numeric memory^f^	1,458	−0.0060	(−0.0192, 0.0072)	0.3718	0.3718	−	1,260	−0.0109	(−0.0302, 0.0085)	0.2706	0.3888	−
	Pairs matching^g^	83,934	1.0027	(1.0022, 1.0032)	2.417e-25	6.714e-25	−	72,440	1.0010	(1.0003, 1.0018)	0.0058	0.0290	−
	Prospective memory^h^	86,326	0.9887	(0.9872, 0.9901)	2.074e-53	2.592e-52	−	74,076	1.0023	(1.0000, 1.0046)	0.0458	0.1145	+
NO_x_ (2010)	Reasoning^c^	83,364	−0.0076	(−0.0086, −0.0066)	1.895e-49	1.184e-48	−	72,106	−0.0015	(−0.0027, −0.0002)	0.0183	0.0556	−
	Reaction time^d^	85,779	1.0005	(1.0004, 1.0006)	7.818e-23	1.777e-22	−	73,683	1.0002	(1.0001, 1.0003)	0.0037	0.0231	−
	Numeric memory^f^	1,458	−0.0042	(−0.0092, 0.0007)	0.0944	0.1124	−	1,260	−0.0073	(−0.0135, −0.0012)	0.0200	0.0556	−
	Pairs matching^g^	83,934	1.0016	(1.0012, 1.0019)	2.149e-17	3.582e-17	−	72,440	1.0011^k^	(1.0002, 1.0020)	0.0187	0.0556	−
	Prospective memory^h^	86,326	0.9919	(0.9909, 0.9929)	3.304e-58	8.260e-57	−	74,076	0.9985	(0.9970, 1.0000)	0.0529	0.1202	−

+/−, point estimate indicates that higher values of the pollutant are associated with better (+) or worse (−) cognitive performance; CI, confidence interval; FDR, false discovery rate; NO_2_, nitrogen dioxide; NO_x_, nitrogen oxides; PM, particulate matter.

All estimates are per unit (μg/m^3^) difference in the air pollutant.

^a^Adjusted for baseline age, gender, ethnic group, Townsend deprivation score, education, smoking status, physical activity, time outdoors, proximity to nearest major road, traffic intensity on nearest major road, and population density category. Adjusted results are reported from models without an interaction term between air pollutant and time outdoors, unless otherwise noted in the table.

^b^Probability adjusted using the Simes-Benjamini-Hochberg method implemented in the Stata qqvalue package.

^c^Linear regression; estimates reported as unstandardized coefficients; possible score range 0 to 13; lower is worse.

^d^Linear regression using log-transformed values; exponentiated estimates reported as rate ratios; values above 1 indicate relatively longer reaction time.

^e^Interaction between PM_10_ and time outdoors: estimates stratified by quintile of time outdoors ranged between 0.9988 (0.9973, 1.0002) in quintile 4 and 1.0011 (0.9997, 1.0026) in quintile 5.

^f^Linear regression; estimates reported as unstandardized coefficients; possible score range 2 to 12; lower is worse.

^g^Negative binomial regression; estimates reported as rate ratios; values above 1 indicate relatively more errors.

^h^Logistic regression; estimates reported as odds ratios; values below 1 indicate relatively lower odds of a correct response.

^i^Interaction between PM_2.5 to 10_ and time outdoors: estimates stratified by quintile of time outdoors ranged between −0.1674 (−0.4992, 0.1643) in quintile 2 and 0.1495 (−0.1705, 0.4696) in quintile 5.

^j^Interaction between NO_2_ and time outdoors: estimates stratified by quintile of time outdoors ranged between 0.9998 (0.9993, 1.0003) in quintile 4 and 1.0005 (1.0001, 1.0010) in quintile 5.

^k^Interaction between NO_x_ and time outdoors: estimates stratified by quintile of time outdoors ranged between 0.999996 (0.9991, 1.0009) in quintile 1 and 1.0011 (1.0001, 1.0022) in quintile 3.

In the adjusted models, the point estimates attenuated towards the null and in some models they reversed direction; the confidence intervals remained narrow but included the null in the majority of models. Five of 25 results had false discovery rate (FDR)-adjusted *p* values below 0.05, but two of these indicated a positive relationship, such that higher pollutant exposure was associated with better performance on the reasoning test. Of the three inverse associations with cognitive performance, two were on the reaction time test; Table 2 shows coefficients per unit difference, which in terms of 1 IQR difference indicated that PM_2.5_ was associated with 0.35% slower reaction time (95% confidence interval [CI]: 0.13, 0.57), and 1 IQR difference in NO_x_ was associated with 0.29% slower reaction time (95% CI: 0.15, 0.44). One IQR difference in NO_2_ was associated with 1.38% higher rate of errors on the pairs matching visual memory test (95% CI: 0.41, 2.50). Interaction tests provided little evidence of a moderating influence of time outdoors.

When noise pollution was added to the adjusted models, the results were virtually unchanged (Supplementary Table S1a). Three estimates with an FDR-adjusted *p* value slightly above alpha in the main models now had adjusted *p* values that were slightly below alpha, although the point estimates and CIs were essentially unchanged compared with the adjusted models that did not include noise pollution. These estimates indicated that 1 IQR difference in PM_2.5 to 10_ was associated with a numeric memory score that was 0.16 points lower (95% CI: −0.29, −0.03; possible score range 2 to 12). One IQR difference in NO_x_ was associated with a reasoning score that was 0.022 points lower (95% CI: −0.041, −0.004; possible score range 0 to 13) and with a numeric memory score that was 0.12 points lower (95% CI: −0.21, −0.02).

### Association between air pollution exposure and change in cognitive function

Table 3 shows the results of the separate regression models for each air pollution exposure and score change on each cognitive test between baseline and follow-up. Samples sizes for these models were much smaller than for the cross-sectional models (*n* = 2,875 to 2,910 unadjusted and *n* = 2,590 to 2,605 with adjustment), and confidence intervals were consequently wider. All of the FDR-adjusted *p* values were above 0.05, and the direction of association indicated by the point estimates was inconsistent. There was no evidence of interaction between the air pollutant measures and time outdoors in any of the models. The results were very similar when noise pollution was added to the adjusted models (Supplementary Table S1b).

**Table 3:** Results of regression models for change in cognitive scores in UK Biobank participants between 2010 and 2012–2013, using centrally-linked pollutant data.

Exposure (year)	Cognitive score	Unadjusted						Adjusted^a^					
	change	*n*	Estimate	(95% CI)	*p* (uncorr)	*p* (FDR)^b^	+/−	*n*	Estimate	(95% CI)	*p* (uncorr)	*p* (FDR)^b^	+/−
PM_10_ (2007)	Reasoning^c^	2,878	0.0088	(−0.0193, 0.0369)	0.5410	0.7213	+	2,593	0.0174	(−0.0217, 0.0565)	0.3824	0.6373	+
	Reaction time^d^	2,896	−1.6117	(−3.2010, −0.0224)	0.0469	0.4450	+	2,605	−3.1616	(−5.5149, −0.8083)	0.0085	0.1270	+
	Pairs matching^e^	2,876	0.0557	(−0.0052, 0.1167)	0.0731	0.4450	−	2,591	0.0324	(−0.0584, 0.1233)	0.4843	0.7451	−
	Prospective memory^f^	2,910	1.0615	(0.9819, 1.1475)	0.1335	0.4450	−	2,597	1.0648	(0.9342, 1.2136)	0.3469	0.6307	−
PM_2.5 to 10_ (2010)	Reasoning^c^	2,877	−0.0372	(−0.1067, 0.0322)	0.2932	0.6516	−	2,592	−0.0910	(−0.2019, 0.0199)	0.1079	0.4760	−
	Reaction time^d^	2,895	−1.8676	(−5.7511, 2.0159)	0.3459	0.6572	+	2,604	3.4625	(−3.0636, 9.9885)	0.2984	0.5968	−
	Pairs matching^e^	2,875	0.0632	(−0.0762, 0.2026)	0.3744	0.6572	−	2,590	−0.0568	(−0.3175, 0.2039)	0.6695	0.8923	+
	Prospective memory^f^	2,909	0.9726	(0.7856, 1.2043)	0.7991	0.9232	+	2,596	0.9940	(0.6954, 1.4210)	0.9738	0.9767	+
PM_2.5_ (2010)	Reasoning^c^	2,877	−0.0044	(−0.0691, 0.0602)	0.8928	0.9232	−	2,592	0.0013	(−0.0860, 0.0886)	0.9767	0.9767	+
	Reaction time^d^	2,895	−3.0284	(−6.7852, 0.7284)	0.1141	0.4450	+	2,604	−6.2530	(−11.1697, −1.3363)	0.0127	0.1270	+
	Pairs matching^e^	2,875	0.0790	(−0.0605, 0.2184)	0.2671	0.6516	−	2,590	−0.0383	(−0.2428, 0.1663)	0.7138	0.8923	+
	Prospective memory^f^	2,909	1.0841	(0.9232, 1.2731)	0.9232	0.9232	−	2,596	1.0107	(0.7685, 1.3293)	0.9393	0.9767	−
NO_2_ (2005)	Reasoning^c^	2,878	0.0041	(−0.0053, 0.0135)	0.3943	0.6572	+	2,593	0.0112	(−0.0038, 0.0261)	0.1443	0.4760	+
	Reaction time^d^	2,896	−0.1386	(−0.6848, 0.4075)	0.6189	0.7736	+	2,605	−0.5876	(−1.4546, 0.2795)	0.1841	0.4760	+
	Pairs matching^e^	2,876	0.0079	(−0.0133, 0.0291)	0.4662	0.7172	−	2,591	−0.0252	(−0.0620, 0.0116)	0.1789	0.4760	+
	Prospective memory^f^	2,910	1.0280	(1.0023, 1.0545)	0.0328	0.4450	−	2,597	1.0326	(0.9868, 1.0806)	0.1657	0.4760	−
NO_x_ (2010)	Reasoning^c^	2,878	0.0003	(−0.0049, 0.0056)	0.8968	0.9232	+	2,593	0.0015	(−0.0056, 0.0087)	0.6720	0.8923	+
	Reaction time^d^	2,896	−0.1005	(−0.3962, 0.1952)	0.5053	0.7213	+	2,605	−0.2785	(−0.6955, 0.1384)	0.1904	0.4760	+
	Pairs matching^e^	2,876	0.0078	(−0.0035, 0.0191)	0.1753	0.5009	−	2,591	−0.0007	(−0.0171, 0.0157)	0.9342	0.9767	+
	Prospective memory^f^	2,910	1.0094	(0.9982, 1.0207)	0.0997	0.4450	−	2,597	1.0098	(0.9927, 1.0272)	0.2623	0.5829	−

+/−, point estimate indicates that higher values of the pollutant are associated with improvement (+) or decline (−) in cognitive performance; CI, confidence interval; FDR; false discovery rate; NO_2_, nitrogen dioxide; NO_x_, nitrogen oxides; PM, particulate matter.

All estimates are per unit (μg/m^3^) difference in the air pollutant.

^a^Adjusted for baseline age, gender, ethnic group, Townsend deprivation score, education, smoking status, physical activity, time outdoors, proximity to nearest major road, traffic intensity on nearest major road, population density category, and time between baseline and follow-up. Adjusted results are reported from models without an interaction term between air pollutant and time outdoors; likelihood ratio test results indicated that the interaction term did not improve model fit.

^b^Probability adjusted using the Simes-Benjamini-Hochberg method implemented in the Stata qqvalue package.

^c^Linear regression; estimates reported as unstandardized coefficients; negative change score values indicate worse performance at follow-up.

^d^Linear regression; estimates reported as unstandardized coefficients; positive change score values indicate slower performance at follow-up.

^e^Linear regression; estimates reported as unstandardized coefficients; positive change score values indicate more errors at follow-up.

^f^Logistic regression; estimates reported as odds ratios; values above 1 indicate relatively higher odds of performance decline at follow-up.

### Sensitivity analyses

#### Impact of prevalent neurological disorders

At baseline, 3,642 (4.2%) of the study population (i.e. those assessed on or after 01 January 2010) self-reported a condition that may affect brain function. When these participants were excluded from the analyses, the results of the cross-sectional models were virtually unchanged (Supplementary Table S2a) compared with the main models (Table 2). The results of the follow-up models excluding these participants (Supplementary Table S2b) followed the same overall pattern as the main models (Table 3), although with minor variation in point estimates and confidence interval limits.

#### Missing covariate data

Across all models, up to 12,249 (14.2%) of the participants did not have complete covariate data for the adjusted cross-sectional analyses, and up to 293 (10.1%) did not have complete covariate data for the follow-up analyses. When the unadjusted cross-sectional models were re-run using only participants with complete covariate data (Supplementary Table S3a), all the point estimates attenuated slightly towards the null, but this did not account for the differences seen between the unadjusted and adjusted results in the main models (Table 2). The results of the unadjusted follow-up models in participants with complete covariate data (Supplementary Table S3b) showed variation in the point estimates compared with the main models, both towards and away from the null, but this did not alter the interpretation of the adjusted main model results presented in Table 3.

#### Air pollution data source

Supplementary Table S4a shows that the median pollutant levels, as measured by Defra and mapped to each participant’s address in the relevant year, were approximately 12–25% lower than those recorded in the central UK Biobank dataset using the baseline address. An exception was the PM_2.5_ measure, which was 23% higher in the Defra dataset. As with the centrally-recorded data, the median pollutant levels recorded by Defra were lower in the follow-up sample than in the baseline sample.

The results were similar when the main cross-sectional analyses were repeated using the Defra data as the independent variables (Table 4), with the unadjusted analyses again indicating small associations between higher pollutant levels and worse performance on most cognitive tests. Eight of the 25 adjusted models had FDR-adjusted *p* values below 0.05, all of which indicated an inverse relationship between pollutant levels and cognitive performance. The largest effect sizes were seen for NO_x_ exposure, for which 1 IQR difference was associated with: 2.92% (95% CI: 1.24, 4.62) higher rate of errors on the pairs matching memory test; 0.58 (95% CI: −0.96, −0.19) lower score on the numeric memory test (possible score range 2 to 12); and 0.21% (95% CI: 0.00, 0.41) slower reaction time. One IQR difference in PM_2.5 to 10_ was associated with 0.04 (95% CI: 0.02, 0.06) lower score on the reasoning test (possible score range 0 to 13). Three of these models showed evidence of interaction, as detailed in Table 4: for each, the inverse relationship between pollutant level and cognitive performance was strongest in participants in the middle quintile of time spent outdoors.

**Table 4:** Results of regression models for cognitive performance in UK Biobank participants at baseline (2010), using Defra pollutant data.

Exposure(year)	Cognitive score	Unadjusted						Adjusted^a^					
		*n*	Estimate	(95% CI)	*p* (uncorr)	*p* (FDR)^b^	+/−	*n*	Estimate	(95% CI)	*p* (uncorr)	*p* (FDR)^b^	+/−
PM_10_ (2007)	Reasoning^c^	83,235	−0.0175	(−0.0218, −0.0133)	4.552e-16	6.322e-16	—	71,163	0.0052	(0.0001, 0.0104)	0.0475	0.1080	+
	Reaction time^d^	85,556	1.0017	(1.0013, 1.0021)	6.988e-18	1.092e-17	−	72,670	0.9997^e^	(0.9988, 1.0005)	0.4756	0.6606	+
	Numeric memory^f^	1,474	−0.0307	(−0.0880, 0.0265)	0.2924	0.3178	−	1,257	−0.0393	(−0.1156, 0.0371)	0.3134	0.5352	−
	Pairs matching^g^	83,783	1.0086	(1.0070, 1.0102)	5.090e-26	1.273e-25	−	71,489	1.0035	(1.0015, 1.0056)	0.0009	0.0075	−
	Prospective memory^h^	86,079	0.9690	(0.9646, 0.9735)	7.851e-41	3.271e-40	−	73,039	1.0070	(1.0003, 1.0138)	0.0413	0.1033	+
PM_2.5 to 10_ (2010)	Reasoning^c^	84,496	−0.1481	(−0.1680, −0.1283)	2.650e-48	2.208e-47	−	72,106	−0.0431	(−0.0638, −0.0225)	4.263e-5	0.0011	−
	Reaction time^d^	86,955	1.0075	(1.0056, 1.0093)	8.196e-16	1.078e-15	−	73,683	1.0014^i^	(0.9976, 1.0053)	0.4725	0.6606	−
	Numeric memory^f^	1,478	−0.0076	(−0.1005, 0.0853)	0.8725	0.8725	−	1,260	0.0145	(−0.0806, 0.1096)	0.7648	0.8313	+
	Pairs matching^g^	85,068	1.0281	(1.0206, 1.0356)	1.288e-13	1.610e-13	−	72,440	1.0037	(0.9952, 1.0123)	0.3910	0.6109	−
	Prospective memory^h^	87,508	0.8733	(0.8553, 0.8916)	2.246e-37	0.021e-37	−	74,076	1.0073	(0.9803, 1.0351)	0.5990	0.7488	+
PM_2.5_ (2010)	Reasoning^c^	84,496	−0.0493	(−0.0575, −0.0411)	5.431e-32	1.697e-31	−	72,106	0.0022	(−0.0077, 0.0121)	0.6595	0.7851	+
	Reaction time^d^	86,955	1.0035	(1.0028, 1.0043)	1.423e-20	2.372e-20	−	73,683	0.9995^j^	(0.9979, 1.0011)	0.5798	0.7488	+
	Numeric memory^f^	1,478	−0.0624	(−0.1455, 0.0207)	0.1412	0.1605	−	1,260	−0.0491	(−0.1460, 0.0479)	0.3211	0.5352	−
	Pairs matching^g^	85,068	1.0160	(1.0129, 1.0191)	5.294e-25	1.203e-24	−	72,440	1.0053	(1.0014, 1.0092)	0.0082	0.0342	−
	Prospective memory^h^	87,508	0.9380	(0.9298, 0.9463)	3.849e-46	2.406e-45	−	74,076	1.0153	(1.0024, 1.0284)	0.0192	0.0533	+
NO_2_ (2005)	Reasoning^c^	82,356	−0.0110	(−0.0131, −0.0090)	3.556e-26	9.878e-26	−	70,457	0.0004	(−0.0020, 0.0029)	0.7263	0.8253	+
	Reaction time^d^	84,608	1.0008	(1.0006, 1.0010)	8.842e-18	1.300e-17	−	71,924	1.0000^k^	(0.9996, 1.0004)	0.9270	0.9270	NA
	Numeric memory^f^	1,458	−0.0017	(−0.0159, 0.0125)	0.8179	0.8520	−	1,241	−0.0406 ^l^	(−0.0696, −0.0116)	0.0060	0.0300	−
	Pairs matching^g^	82,897	1.0036	(1.0029, 1.0044)	5.775e-21	1.031e-20	−	70,776	1.0012	(1.0002, 1.0022)	0.0139	0.0434	−
	Prospective memory^h^	85,111	0.9845	(0.9823, 0.9866)	2.707e-44	1.354e-43	−	72,283	1.0017	(0.9985, 1.0049)	0.3069	0.5352	+
NO_x_ (2010)	Reasoning^c^	84,496	−0.0080	(−0.0089, −0.0071)	2.684e-64	6.710e-63	−	72,106	−0.0018 ^m^	(−0.0038, 0.0002)	0.0736	0.1533	−
	Reaction time^d^	86,955	1.0004	(1.0004, 1.0005)	7.028e-25	1.464e-24	−	73,683	1.0001	(1.0000, 1.0002)	0.0119	0.0425	−
	Numeric memory^f^	1,478	−0.0068	(−0.0149, 0.0012)	0.0966	0.1150	−	1,260	−0.0280^n^	(−0.0466, −0.0094)	0.0032	0.0200	−
	Pairs matching^g^	85,068	1.0017	(1.0013, 1.0020)	6.678e-22	1.284e-21	−	72,440	1.0014^o^	(1.0006, 1.0022)	0.0004	0.0050	−
	Prospective memory^h^	87,508	0.9921	(0.9911, 0.9930)	1.132e-56	1.415e-55	−	74,076	1.0001	(0.9987, 1.0015)	0.8885	0.9255	+

+/−, point estimate indicates that higher values of the pollutant are associated with better (+) or worse (−) cognitive performance; CI, confidence interval; FDR, false discovery rate; NA, not applicable; NO_2_, nitrogen dioxide; NO_x_, nitrogen oxides; PM, particulate matter.

All estimates are per unit (μg/m^3^) difference in the air pollutant.

^a^Adjusted for baseline age, gender, ethnic group, Townsend deprivation score, education, smoking status, physical activity, time outdoors, proximity to nearest major road, traffic intensity on nearest major road, and population density category. Adjusted results are reported from models without an interaction term between air pollutant and time outdoors, unless otherwise noted in the table.

^b^Probability adjusted using the Simes-Benjamini-Hochberg method implemented in the Stata qqvalue package.

^c^Linear regression; estimates reported as unstandardized coefficients; possible score range 0 to 13; lower is worse.

^d^Linear regression using log-transformed values; exponentiated estimates reported as rate ratios; values above 1 indicate relatively longer reaction time.

^e^Interaction between PM_10_ and time outdoors: estimates stratified by quintile of time outdoors ranged between 0.9989 (0.9977, 1.0002) in quintile 4 and 1.0008 (0.9995, 1.0020) in quintile 5.

^f^Linear regression; estimates reported as unstandardized coefficients; possible score range 2 to 12; lower is worse.

^g^Negative binomial regression; estimates reported as rate ratios; values above 1 indicate relatively more errors.

^h^Logistic regression; estimates reported as odds ratios; values below 1 indicate relatively lower odds of a correct response.

^i^Interaction between PM_2.5 to 10_ and time outdoors: estimates stratified by quintile of time outdoors ranged between 0.9940 (0.9887, 0.9992) in quintile 4 and 1.0036 (0.9986, 1.0086) in quintile 5.

^j^Interaction between PM_2.5_ and time outdoors: estimates stratified by quintile of time outdoors ranged between 0.9979 (0.9954, 1.0003) in quintile 4 and 1.0015 (0.9991, 1.0038) in quintile 5.

^k^Interaction between NO_2_ and time outdoors: estimates stratified by quintile of time outdoors ranged between 0.9992 (0.9986, 0.9999) in quintile 4 and 1.0005 (0.9999, 1.0011) in quintile 5.

^l^Interaction between NO_2_ and time outdoors: estimates stratified by quintile of time outdoors ranged between −0.0491 (−0.0862, −0.0121) in quintile 3 and 0.0459 (0.0060, 0.0859) in quintile 5.

^m^Interaction between NO_x_ and time outdoors: estimates stratified by quintile of time outdoors ranged between −0.0054 (−0.0083, −0.0026) in quintile 4 and 0.0007 (−0.0020, 0.0033) in quintile 5.

^n^Interaction between NO_x_ and time outdoors: estimates stratified by quintile of time outdoors ranged between −0.0323 (−0.0556, −0.0089) in quintile 3 and 0.0235 (−0.0080, 0.0550) in quintile 2.

^o^Interaction between NO_x_ and time outdoors: estimates stratified by quintile of time outdoors ranged between 0.9998 (0.9988, 1.0008) in quintile 5 and 1.0016 (1.0007, 1.0025) in quintile 3.

The follow-up models were also repeated using the Defra data (Supplementary Table S4b). As with the analyses using the centrally-linked pollutant data, all of the FDR-adjusted *p* values were above 0.05, and there were no interactions between the air pollutant measures and time outdoors in any of the models.

## Discussion

In this large sample of adults from the UK Biobank general population cohort, cross-sectional associations between air pollutant exposure and cognitive performance attenuated towards the null after adjustment for important confounders. Following adjustment, estimated associations were inconsistent in direction and of very small magnitude. The estimates with FDR-adjusted *p* values below 0.05 indicated that 1 IQR higher air pollutant exposure was associated on average with 0.35% slower reaction time (95% CI: 0.13, 0.57), a 2.92% higher error rate on the pairs matching visuospatial memory test (95% CI: 1.24, 4.62), and numeric memory scores that were 0.58 points lower (95% CI: −0.96, −0.19). There was little evidence that the results varied substantially by self-reported time spent outdoors. Sensitivity analyses showed slightly stronger associations when linkage using an alternative source of air pollutant data took account of participants’ address history. Follow-up analyses of cognitive change scores did not detect any association between pollutant exposure and magnitude of score change; the precision of these results was reduced as a consequence of the smaller sample size, thus decreasing the power to detect small associations reliably. The results of both cross-sectional and follow-up analyses were robust to the potential influence of prevalent neurological disorders and missing covariate data. Overall, this study indicated that the association between air pollution exposure and cognitive performance was at most very weak in this population, particularly when confounding factors were taken into account.

The principal strength of this study was the sample size, which for the cross-sectional analyses was at least five-fold larger than similar studies in the literature^19–28^, thus allowing associations to be estimated with much greater precision than before. The study also benefited from the availability of multiple pollutant measures and multiple cognitive tests, measured in the same manner for all participants. Important covariates were also measured consistently, and adjusted analyses were planned in a principled way based on graphical analysis of assumed inter-relationships between variables. It was possible to investigate the potentially moderating relationship between neighbourhood pollutant measures and individual-level data regarding time spent outdoors, which had not been addressed in the previous literature.

A number of limitations must be considered. In common with most previous studies in this field, individual-level pollutant measures were not available, but efforts were made to mitigate this by analysing data regarding time spent outdoors (albeit that this measure was self-reported and did not necessarily represent time spent outdoors in the neighbourhood of residence from which the pollution measures were taken). The air pollution measures were also taken from different years, up to five years prior to cognitive assessment. The cognitive tests were brief and were subject to measurement error; the previously-noted low reliability of some of the tests (e.g. pairs matching test-retest correlation <0.2)^39^ is likely to have biased the change score analyses towards the null, particularly in the relatively short follow-up duration studied here. It is possible to conduct joint analyses of the cognitive outcome measures using latent factors or multivariate modelling (e.g. canonical correlation analysis), which would address the problem of low reliability of individual measures, but this would also reduce the ability to detect any domain-specific relationships that may exist, e.g. affecting memory tasks only. The temporal order of some of the measures was unclear: despite the restrictions imposed on the timing of the pollution measures relative to the date of the baseline cognitive assessment, it was not possible to tell when participants’ cognitive performance reached the level at which it was measured at baseline, because no premorbid cognitive estimates were available. It is therefore possible that cognitive decline had occurred prior to the study period, which in turn may or may not have been causally linked to air pollution exposure at an earlier age. Limitations regarding temporal relationships also apply to the graphical model used to inform the model adjustments (see Supplementary Methods). It should be noted that the UK Biobank cohort had a low opt-in rate and it is not representative of the general UK population in some respects^40^, and this may lead to biased association estimates if the exposure and outcome have together influenced participation^41^. Non-representativeness was further amplified among the sub-group that returned for the follow-up visit, as shown in Table 1 here. Furthermore, UK Biobank recruited adults aged 40 to 70 years, and so the results of this study may not be generalisable beyond this age range.

Previous studies had reported mixed evidence regarding associations between air pollutants and cognitive test performance, with estimates varying in magnitude and/or predicated on very large differences in pollutant measures. For example, Ailshire and Clarke^19^ reported an adjusted error rate ratio of 1.53 (95% CI: 1.02, 2.30) in association with higher levels of PM_2.5_ on tests of orientation and working memory, but this was per 10 μg/m^3^ increment in PM_2.5_, which in their sample equated to more than 3 SD (mean 13.8, SD 3.1). The largest previous cross-sectional study, by Zeng, *et al*.^26^, reported that a 1-point increment on an ordinal air pollution score (range 1 = least pollution to 7 = most) was associated with a modest adjusted OR for impaired Mini-Mental State Examination performance (score <18) of 1.09 (95% CI: 1.01, 1.18). Cross-sectional results in the present study indicated very small cognitive performance differences associated with meaningful (1 IQR) sample differences in pollutant exposure levels. In two instances these associations were in a protective direction: analyses using the centrally linked pollutant data (Table 2) showed a very small positive association between reasoning performance and both PM_10_ and NO_2_. Given that these associations were not evident to the same degree in the Defra data analyses (Table 4), and that there is no previous evidence to suggest a protective relationship, these unexpected results may be spurious false positives or may reflect selection bias in the cohort.

Overall, the discrepancy between the present findings and some previously reported cross-sectional results may reflect different sample sizes and approaches to covariate adjustment, as noted earlier. Sample age ranges have also varied across previous studies (mean 37 years^27^ to mean 86 years^24,26^), although there does not appear to be a clear link between previous results and participant age, with cross-sectional studies in both early-middle^27^ and older adulthood^28^ showing similar null findings to ours. There is some evidence of adverse neurodevelopmental and cognitive outcomes in children exposed to high levels of air pollutants^2^, and it would be important to investigate this further in younger cohorts. Most studies have been conducted in the USA^19–21,25,27,28^, with a small number from other countries (Germany^22,23^ and China^24,26^), and it remains unclear to what extent research in countries with stricter emissions regulations and relatively low average pollution levels (such as the UK) are generalisable to other settings.

Fewer studies have examined the relationship between air pollution exposure and change in cognitive test performance over time^29–31^. The largest of these^29^ had a sample size of 20,150 and a follow-up period of 4–5 years, and found no reliable association between fine particulate matter (PM_2.5_) and performance decline on the brief ‘Six Item Screener’ cognitive test: the adjusted OR was 0.98 (95% CI: 0.72, 1.34) per 10 µg/m^3^ increment in PM_2.5_ (equivalent to 4 IQR in their sample). The studies by Tonne, *et al*.^30^ and Weuve, *et al*.^31^ reported a mixed picture, with detrimental associations evident in only some analyses, in cohorts followed for five and two years respectively. Evidence from the present study, with follow-up for 2.8 years (SD 0.2) and a sample size of ~2,600 in adjusted models, was in keeping with previous null results.

The evidence from this and previous studies of cognitive test performance are somewhat at odds with other reports showing consistently increased likelihood of MCI or dementia diagnosis in those with higher air pollution exposure history or closer proximity to major roads^32–37^. A possible causal link between air pollution and dementia was further suggested in a recent study of magnetite nanoparticles in post-mortem brains of a small sample of patients with Alzheimer’s disease^12^. It may be that selection bias is operating in cognitive assessment research, such that participants who are able and willing to enrol and provide data are less at risk of cognitive impairment; such a bias would not affect studies of dementia outcomes that are conducted using routine health records.

Further follow-up of the UK Biobank cohort will provide future opportunities to investigate brain imaging measures, as well as cognitive performance over longer periods and incident dementia outcomes. Ongoing monitoring of participants’ address history and detailed modelling of the built environment^42^ will add valuable information to enhance our understanding of the influence of the local environment on cognitive and other health outcomes.

## Methods

### Participants

Adults aged 40 to 69 years who were registered with the National Health Service (NHS) and living within 25 miles of a study assessment centre were invited by mail to participate in UK Biobank^38^. No exclusion criteria were applied during recruitment. Twenty-two assessment centres were in operation across England, Scotland and Wales at different times between 2006 and 2010. Approximately nine million invitations were issued to achieve the cohort size of ~502,000, indicating an overall response rate of approximately 5.6%^43^. Invitations for a follow-up visit in 2012–2013 were sent by email to 103,514 cohort participants living near the UK Biobank coordinating centre in northwest England, of whom 20,345 (19.7%) attended. All participants gave written informed consent. This study was conducted under generic approval from the NHS National Research Ethics Service (Ref. 11/NW/0382).

The present study population included all participants who attended for baseline assessment on or after 01 January 2010, and who had data on at least one air pollution exposure measure and at least one cognitive test score. This baseline cut-off date was chosen so that the cognitive measures would be from the same or a later year than the physical environment measures (see below). From this study population, those participants who also attended the follow-up visit in 2012–2013 were included in the change score analyses.

### Materials and procedure

Baseline and follow-up assessment visits lasted approximately 2–3 hours, incorporating consent processes, computerised touchscreen questionnaire (including cognitive tests), nurse interview and physical measurements. All assessments were administered in a standardised order, according to a standard operating procedure. Administration and scoring of cognitive tests and questionnaires was automated.

#### Air pollution measures

UK Biobank air pollution data: Air pollution and local environment measures were provided by the Small Area Health Statistics Unit (http://www.sahsu.org/) as part of the BioSHaRE-EU Environmental Determinants of Health Project (http://www.bioshare.eu/), and were linked centrally to the assessment data by UK Biobank analysts (http://biobank.ctsu.ox.ac.uk/crystal/docs/EnviroExposEst.pdf). These measures were modelled at participants’ baseline residential addresses. A total of 7,221 addresses (approximately 1.4%) could not be geo-coded and therefore have missing data. Particulate matter of up to 10 μm diameter (PM_10_, PM_2.5 to 10_ and PM_2.5_), nitrogen dioxide (NO_2_) and total nitrogen oxides (NO_x_) were measured as annual average values in μg/m^3^. Estimates for the years 2005 to 2007 were derived from European Union (EU)-wide air pollution maps (resolution 100 m × 100 m). The X, Y coordinates of participants’ baseline addresses were overlaid on these maps (projected to the British National Grid) and the corresponding air pollution concentration of the 100 m × 100 m grid cell was assigned to the coordinate. These data were from a land use regression (LUR) model for western Europe based on >1500 EuroAirnet monitoring sites, which also included satellite-derived air pollution estimates to improve the model performance^44^. Estimates for the year 2010 were modelled for each address using a LUR model developed as part of the European Study of Cohorts for Air Pollution Effects (ESCAPE; http://www.escapeproject.eu/)^45,46^. ESCAPE estimates for PM in 2010 are valid up to 400 km from the monitoring area (Greater London), but the accuracy of estimates beyond this range (*n* = 33,935 addresses) was unknown and so these were coded as missing within the central UK Biobank dataset. Where a pollutant measure was available for more than one year, data for the earliest available year were analysed, to minimise uncertainty about the temporal order of exposure and outcome. The PM_10_ measure was for 2007; PM_2.5 to 10_ and PM_2.5_ were for 2010; NO_2_ was for 2005; and NO_x_ was for 2010.

Defra air pollution data: The UK Biobank air pollution data described above were mapped to participants’ baseline addresses, without taking account of address history at the time the pollutant measure was recorded. Separate data were made available subsequently by UK Biobank regarding participants’ past address history (east and north coordinates rounded to 1 km), and the date the participant was first recorded at each location. These were used in the present study to map air pollution data provided by the UK Government Department for Environment, Food and Rural Affairs (Defra) (https://uk-air.defra.gov.uk/data/pcm-data). Modelled data^47^ for the same pollutants in the same years as above (PM_10_ for 2007; PM_2.5 to 10_ and PM_2.5_ for 2010; NO_2_ for 2005; and NO_x_ for 2010) were mapped to participants’ addresses in the relevant year. These data were used only in sensitivity analyses, described below.

#### Other local environment measures

Population density (urban/rural) was classified categorically by UK Biobank, by combining each participant’s baseline residential postcode with data generated from the 2001 census, using the GeoConvert tool provided by the UK Data Service Census Support (http://geoconvert.mimas.ac.uk/). Road traffic measures were provided for the year 2008 from the Road Traffic Statistics Branch at the Department for Transport attached to the local road network; traffic data for unmonitored links were estimates based on surrounding monitored links. A major road was defined as a road with traffic intensity >5000 motor vehicles per 24 hours. Traffic intensity on the nearest major road was measured as the average total number of motor vehicles per 24 hours. Proximity to the nearest major road was calculated as the inverse distance (1/m) from the residential location. Data were also available regarding noise pollution, and these were included in supplementary analyses to address potential residual confounding (see *Data analysis* section below). Noise estimates for the year 2009 were modelled using a version of the Common NOise aSSessment methOdS (CNOSSOS-EU) noise model^48,49^; average level of noise pollution in decibels was calculated as a weighted level measured over a 24-hour period, with a 10 decibel penalty added between 23:00 h and 07:00 h.

#### Sociodemographic and lifestyle measures

Age was recorded in whole years. Gender was self-reported as male or female. Self-reported ethnic background was grouped categorically as white, Asian/Asian British, black/black British, Chinese, or mixed/other ethnic group. Neighbourhood-level socioeconomic status was measured using the Townsend index of material deprivation^50^. This was calculated by UK Biobank immediately before the baseline date, based on census data regarding unemployment, car ownership, home ownership and household overcrowding; each participant was assigned a score corresponding to the census output area in which their residential postcode was located, with higher values indicating greater relative deprivation (see Supplementary Methods). Educational qualifications were self-reported, and for the present study were dichotomised according to whether or not participants held a university/college degree. Self-reported smoking status data were used by UK Biobank to categorise participants as current, former or never smokers; these were dichotomised for the present study as ‘ever smoker’ (current or former) versus ‘never smoker’. Physical activity in a typical week was recorded using self-reported items from the International Physical Activity Questionnaire short form^51^, and was converted into a single measure of total physical activity in metabolic equivalent of task (MET) hours per week, weighted by intensity (walking, moderate or vigorous). Participants were asked to estimate how many hours they spent outdoors in a typical day in summer, and in a typical day in winter. Responses were given in whole hours from 0 to 24, and participants whose responses exceeded 10 were asked to check and confirm this. An additional option of ‘Less than an hour a day’ was available, and for the present study was assigned a value of 0.5. Overall time outdoors was calculated as the mean of the hours per day in summer and winter together.

#### Cognitive assessment

The format and psychometric properties of the cognitive tests used in UK Biobank have been described by us previously^39,52^. All tests were administered visually via touchscreen, and scoring was automated. The tasks assessed reasoning (total correct of 13 items), reaction time (mean time in milliseconds to press a button in response to matching cards), numeric memory (longest numeric string recalled in reverse), visuospatial memory (‘pairs matching’ test: total errors when recalling positions of matching cards) and prospective memory (successfully carrying out an instruction after a filled delay). Higher values indicate better performance on the reasoning and numeric memory tests, and worse performance on the reaction time and pairs matching tests. Prospective memory test performance was categorised dichotomously as 1 for a correct response on the first attempt and 0 otherwise. Details of all tests are provided in the Supplementary Methods. The numeric memory test was removed from the UK Biobank baseline assessment battery part-way through recruitment, for reasons of time, resulting in lower sample sizes on this test than on the other four tests.

Change on the cognitive tests was measured by subtracting the baseline score from the follow-up score. Raw scores were used in these calculations, without any replacement of outlying values. Negative change score values on the reasoning test indicate worse performance at follow-up; positive change score values on the reaction time test indicate slower performance at follow-up; positive change score values on the pairs matching test indicate more errors at follow-up. Change on the prospective memory test was dichotomised as ‘worse at follow-up’ = 1, versus ‘same or better at follow-up’ = 0 (i.e., ‘worse at follow-up’ means the participant gave the correct response at baseline and an incorrect response at follow-up). The numeric memory test was not administered at follow-up, so no change scores were available.

### Data analysis

All analyses were performed using Stata version 13^53^. Data were summarised descriptively to characterise the baseline and follow-up samples. Quintiles were derived for the measures of major road proximity, traffic intensity, noise pollution, physical activity and time outdoors, based on all available data in the whole UK Biobank cohort at baseline. Regression models were used to estimate the association between air pollution exposures (independent variable) and cognitive performance (dependent variable), with and without adjustment for other covariates. The pollutant data provided centrally by UK Biobank were used in all primary analyses, and sensitivity analyses were conducted using the Defra data (see below). Multicollinearity between the air pollution measures and the covariates was within acceptable limits (variance inflation factor values 1.77 to 1.96). For all models, normal-approximation 95% confidence intervals (CI) were generated from bootstrapped standard errors (5000 replicates)^54^. Since there were 25 cross-sectional and 20 follow-up regression analyses, *p* values (two-tailed) for the pollutant coefficients were adjusted using the Simes-Benjamini-Hochberg false discovery rate (FDR) method^55^; both unadjusted and FDR-adjusted *p* values are reported. Alpha was 0.05 (FDR-adjusted). ‘Do not know’ and ‘Prefer not to answer’ responses were treated as missing. Missing data were not imputed.

For the cross-sectional analyses, each baseline cognitive measure was regressed separately on each air pollution measure (continuous), firstly in unadjusted models and then with adjustment for baseline age, gender, ethnic group, Townsend score (continuous), education, smoking status, physical activity (quintiles), time outdoors (quintiles), major road proximity (quintiles), traffic intensity (quintiles), and population density category. The Supplementary Methods shows a directed acyclic graph of the assumptions underpinning the analytical model: the aim of covariate adjustment was to minimise confounding influences on the association between air pollution exposure and cognitive performance, rather than to construct a multivariable risk prediction model for cognitive outcome. Linear regression was used for the reasoning and numeric memory scores, which were approximately normally distributed, and unstandardized coefficients are reported. Positive skew in the reaction time distribution was addressed using a natural log transformation, and linear regression was then used; exponentiated results are reported as rate ratios (RR). Outlying pairs matching error count values (>30; *n* = 49, 0.06%) were replaced with a value of 30, and only participants who finished the task (achieved all six pairs) were included in the analyses. The pairs matching error count distribution remained overdispersed; a negative binomial model was used and coefficients are reported as RR. A logistic regression model was used for the prospective memory score, and results are reported as odds ratios (OR).

For the follow-up analyses, each cognitive change measure was regressed separately on each air pollution measure (continuous), firstly in unadjusted models and then with adjustment for duration between baseline and follow-up (continuous) as well as baseline age, gender, ethnic group, Townsend score (continuous), education, smoking status, physical activity (quintiles), time outdoors (quintiles), major road proximity (quintiles), traffic intensity (quintiles), and population density category. Change scores for reasoning, reaction time and pairs matching followed an approximately normal distribution, and were analysed with linear regression; unstandardized coefficients are reported. Change on the prospective memory test was analysed using logistic regression, with results reported as OR. For the pairs matching analyses, only participants who finished the task (achieved all six pairs) at both time points were included.

To test whether the association between neighbourhood air pollution exposure and cognitive outcome varied according to the amount of time typically spent outdoors, all cross-sectional and follow-up models were run with and without a product term (air pollutant measure * time outdoors [quintiles]), and the fit of the model with and without the product term was compared using the likelihood ratio test. It was predicted that associations would be stronger among those who spent the most time outdoors, either because spending time outdoors exposes individuals to more pollution, or because the neighbourhood-level measure would more accurately reflect actual pollutant exposure in those who spent more time outdoors. When the *p* value of the likelihood ratio test was <0.05, separate models were run for each quintile of time outdoors.

In a supplementary analysis, all adjusted models were repeated with noise pollution (quintiles) as an additional covariate. The purpose was to adjust for possible residual confounding from antecedents of both air pollution and noise pollution (see Supplementary Methods).

### Sensitivity analyses

#### Impact of prevalent neurological disorders

To address the possibility that associations between pollution exposure and cognitive function might be driven by participants with prevalent neurological disorders, the main analyses (unadjusted and adjusted) were repeated after excluding participants who had self-reported conditions that affect brain function at baseline (listed in the Supplementary Methods).

#### Missing covariate data

The unadjusted regression models were based on all available data, which meant that differences between unadjusted and adjusted estimates may have been partly due to the inclusion of different participants (all available in the unadjusted model, versus only those with complete covariate data in the adjusted). The unadjusted models were therefore repeated using only participants who had complete covariate data.

#### Air pollution data source

The air pollution exposure data provided by UK Biobank were mapped centrally to participants’ baseline addresses, without taking account of address history in the year in which the pollutant measure was recorded. The Defra data described above were linked to participants’ addresses in the same year as the pollutant was measured, thus potentially reducing measurement error in the exposure. The main cross-sectional and follow-up analyses were repeated with the Defra data as the independent variable, for comparison.

## Electronic supplementary material

Supplementary Information

## Data Availability

UK Biobank is an open access resource. Data are available to bona fide scientists, undertaking health-related research that is in the public good. Access procedures are described at http://www.ukbiobank.ac.uk/using-the-resource/. Defra air pollutant data are freely available for download from https://uk-air.defra.gov.uk/data/pcm-data.
