# Supplementary material for: Cross-sectional and longitudinal analyses of outdoor air pollution exposure and cognitive function in UK Biobank
Source: Sci Rep. 2018 Aug 14;8:12089. doi: 10.1038/s41598-018-30568-6 (PMC6092329; doi:10.1038/s41598-018-30568-6)
Supplement: Supplementary file 1 — Supplementary Information [file 41598_2018_30568_MOESM1_ESM.pdf]

Supplementary Information

Cross-sectional and longitudinal analyses of outdoor air pollution exposure and cognitive function in UK Biobank

Breda Cullen, Danielle Newby, Duncan Lee, Donald M. Lyall, Alejo J. Nevado-Holgado, Jonathan J. Evans, Jill P. Pell, Simon Lovestone, and Jonathan Cavanagh

Contents

Supplementary Methods 2

Supplementary Tables 9

Table S1a Results of regression models for cognitive performance in UK Biobank participants at baseline (2010), including adjustment for noise pollution 9

Table S1b Results of regression models for change in cognitive scores in UK Biobank participants between 2010 and 2012-2013, including adjustment for noise pollution 10

Table S2a Results of regression models for cognitive performance in UK Biobank participants at baseline (2010), excluding participants with conditions affecting brain function 11

Table S2b Results of regression models for change in cognitive scores in UK Biobank participants between 2010 and 2012-2013, excluding participants with conditions affecting brain function at baseline 13

Table S3a Comparison of unadjusted regression models for cognitive performance in UK Biobank participants at baseline (2010), using all available participants versus only participants with complete covariate data 14

Table S3b Comparison of unadjusted regression models for change in cognitive scores in UK Biobank participants between 2010 and 2012-2013, using all available participants versus only participants with complete covariate data 15

Table S4a Summary of Defra pollution data used in sensitivity analyses 16

Table S4b Results of regression models for change in cognitive scores in UK Biobank participants between 2010 and 2012-2013, using Defra pollutant data 17

Supplementary References 18

Supplementary Methods

Reporting follows STrengthening the Reporting of OBservational studies in Epidemiology (STROBE) guidelines [1].

Townsend Index

The Townsend Index is an area-based measure of material deprivation, based on four variables: unemployment (as a percentage of those aged 16 and over who are economically active); non-car ownership (as a percentage of all households); non-home ownership (as a percentage of all households); and household overcrowding (households with >1 person per room, as a percentage of all households). The unemployment and overcrowding variables are log-transformed, and then all four variables are converted to z-scores. The overall index score is the sum of the four z-scores. Positive scores represent higher deprivation and negative scores represent lower deprivation, relative to the mean of 0. Details of the method of calculation can be found here: https://census.ukdataservice.ac.uk/get-data/related/deprivation

Cognitive assessment

The tests were designed specifically for UK Biobank but share some characteristics with other established tests of cognitive function. Two tests were included in the protocol throughout the UK Biobank baseline and follow-up phases (reaction time and pairs matching); two tests were introduced in the final two years of baseline recruitment and were retained for the follow-up visit (reasoning and prospective memory); one test was introduced in the final two years of baseline recruitment and was then subsequently removed from later baseline visits and the follow-up visits for reasons of time (numeric memory). Sample size therefore varied across tests, at both baseline and follow-up timepoints. The total time to complete all five tests was approximately 15 minutes.

<u>Reasoning</u>
Thirteen questions were presented sequentially via touchscreen on a self-paced basis with an overall time limit of two minutes. Responses were selected from a multiple-choice array. Any questions not attempted during the two-minute time limit were scored as zero. The score for analysis was an unweighted total from 0 to 13 (UK Biobank data field 20016, known as the 'fluid intelligence' test), with higher scores indicating better performance.

<u>Reaction time</u>
This test was based on a 'Snap'-style computer game, in which participants were asked to press a button with their dominant hand as quickly as possible each time a matching pair of symbols was presented on-screen. Five practice trials were administered, followed by seven test trials. The score for analysis was the mean time (in milliseconds) to press the button, derived from the four trials in which a matching pair occurred (UK Biobank data field 20023). Higher scores indicate slower (i.e. worse) performance.

<u>Numeric memory</u>
A string of numbers was presented on-screen, and after a brief delay participants were asked to enter it from memory, in reverse order, via a numeric keypad. Each string was presented on screen for 2000ms, plus an additional 500ms multiplied by the string length. A delay of 3000ms occurred between clearing the screen and activating the response keypad. All participants began with a string length of two, and successive strings increased by one, up to a maximum string length of 12. The test was discontinued after five successive incorrect responses at a string length of two, or after two successive incorrect responses at string lengths of three or more. The score for analysis was the maximum string length recalled correctly (UK Biobank data field 4282), with higher scores indicating better performance.

<u>Pairs matching</u>
Symbol cards were presented on-screen in a random array. Participants were asked to memorise the position of as many matching pairs as possible. The cards were then turned face down on the screen and participants were asked to touch as many matching pairs as possible in the fewest tries. The score for analysis was the number of errors made while attempting to select the pairs, with a higher score indicating worse performance. Two trials of this task

were administered, one with three pairs of symbols and one with six pairs. Because there was a ceiling effect on the three-pair trial, only the score on the six-pair trial of the test was analysed in the present study (UK Biobank data field 399.0.2).

Prospective memory

The following instruction appeared on the touchscreen: "At the end of the games we will show you four coloured symbols and ask you to touch the blue square. However, to test your memory, we want you to actually touch the orange circle instead". After a delay during which participants completed the other cognitive tasks described above, a screen appeared showing four coloured shapes with the instruction to touch the blue square. If the participant touched the orange circle, their response was recorded as 'correct on first attempt'. If they touched the blue square, they were given a prompt on-screen to try to recall what the original instruction was, and were asked to respond again. If they correctly selected the orange circle after receiving this prompt, their response was recorded as 'correct on second attempt'. All other responses (including no response) were recorded on the system as incorrect. For the present analyses, data were dichotomised as either 'correct on first attempt' or not (derived from UK Biobank data field 20018).

Data analysis

The regression models included covariates chosen to minimise confounding influences on the association between air pollution exposure and cognitive performance. The assumptions underpinning the analytical model were depicted in a directed acyclic graph, and DAGitty software [2] was used to derive an appropriate covariate adjustment set. DAGitty uses an algorithm to determine whether adjusting for certain covariates will block confounding paths in the graph, based on the directional relationships that are depicted between the nodes in the graph. The algorithm uses the rules of 'd-separation' [3]; it is based solely on the structure of the graph, not on any measured data. Supplementary Figure S1a below shows the variables (nodes) and their putative inter-relationships (arrows) that are assumed to give rise to the predicted association between neighbourhood air pollution exposure and cognitive performance; paths representing potential confounding influences are shown in red. Given these assumptions, the DAGitty algorithm determined a minimum sufficient adjustment set to block these confounding paths (Supplementary Figure S1b; blocked confounding paths shown in black). Ethnic group was not part of the minimum adjustment set, but was included as a covariate in the main analyses in this study because of its inclusion in other key studies in the literature; adding this to the adjustment set did not re-introduce bias according to the graph assumptions (Supplementary Figure S1c: main analysis model). Noise pollution was also added to the covariate adjustment set in supplementary analyses: although the graph structure indicated that confounding paths that included noise pollution should already be blocked following adjustment for deprivation, population density, road proximity, traffic intensity and time outdoors (Supplementary Figure S1c), the addition of noise pollution to the covariate adjustment set (Supplementary Figure S1d) may reduce additional residual confounding.

Note that the relationships depicted in the graph are cross-sectional and therefore static, and they do not illustrate the complex time-dependent effects that may be at play. For example, current general health status was assumed in the graph to affect time outdoors and thereby pollution exposure, but pollution exposure (and its antecedents) in the more distant past are likely to have affected general health status over time. Similarly, in some circumstances air pollution levels influence the amount of time that people choose to spend outdoors, and reversing this arrow in the graph would alter the implications for covariate adjustment.

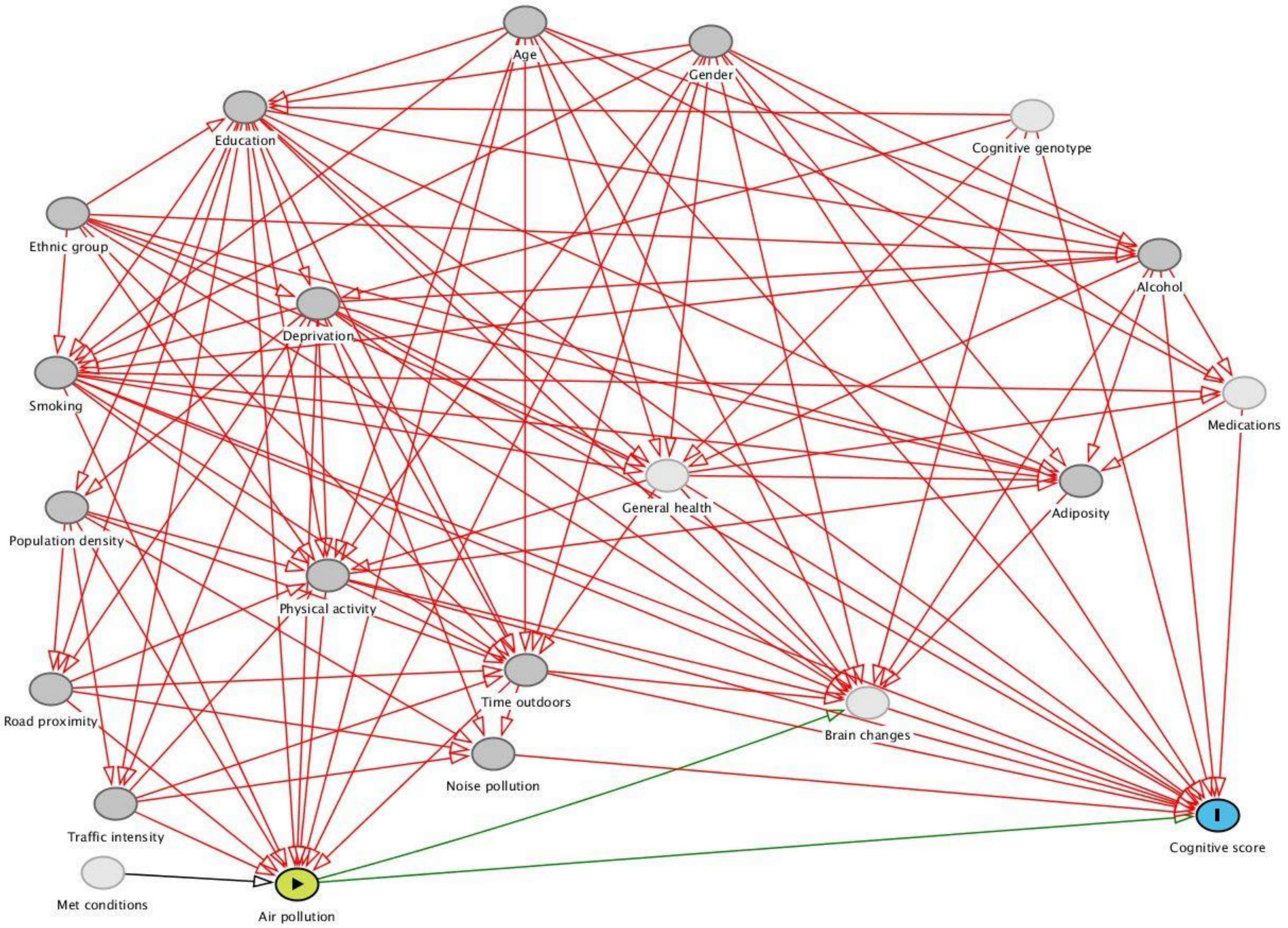

Figure S1a Directed acyclic graph showing the model assumptions.
Nodes represent variables and arrows represent directional relationships between them, based on assumed cross-sectional relationships at the time of the UK Biobank baseline assessment. Light grey nodes are variables that are assumed to directionally influence other nodes, but which were set as unmeasured (latent) in the present study and were therefore not included as candidate covariates for adjustment by the DAGitty algorithm. Green arrows represent assumed causal relationships between air pollutant exposure and cognitive performance. Red arrows represent implied confounding paths before any covariate adjustment has been applied.
Met = meteorological.

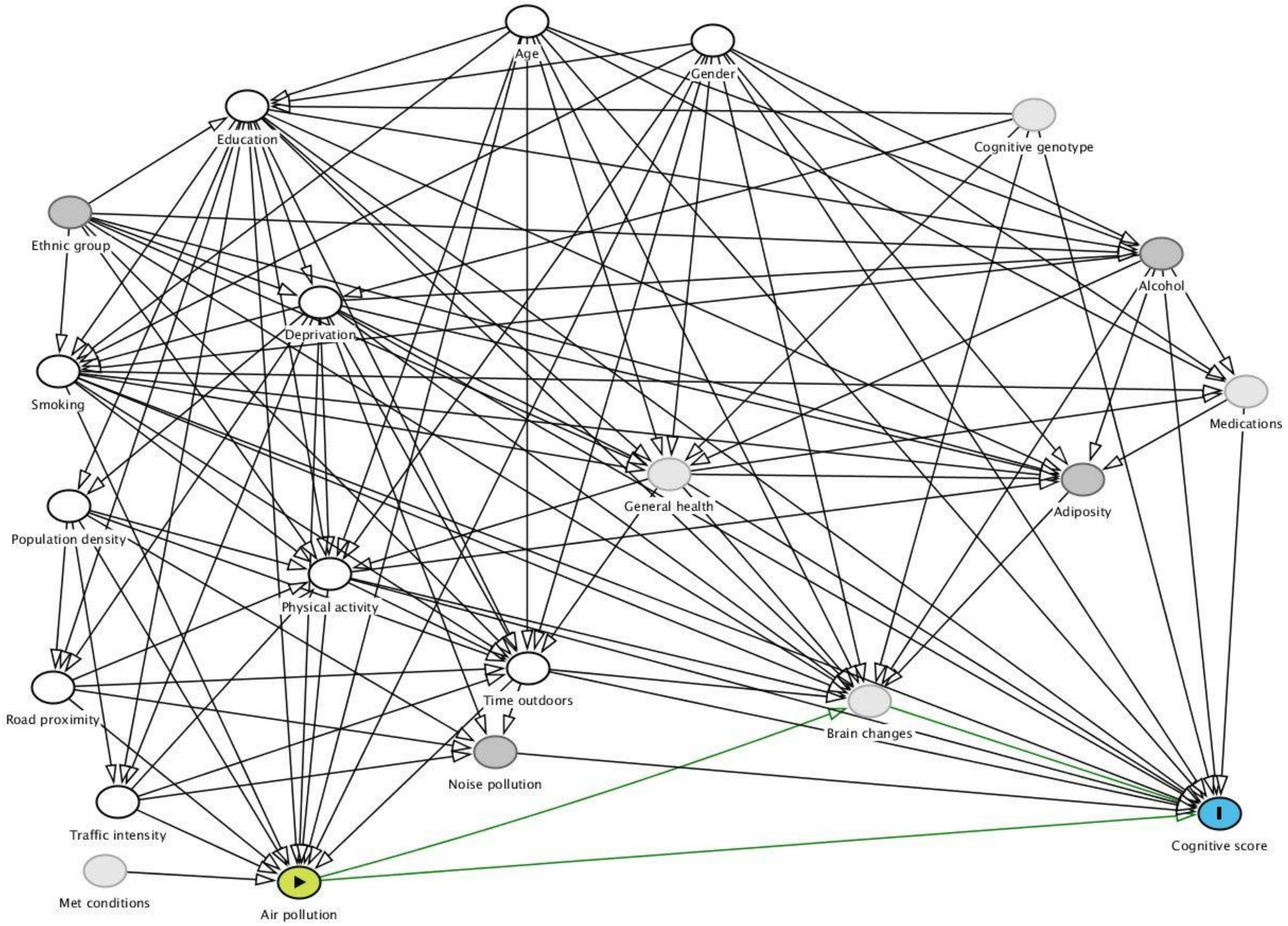

Figure S1b Directed acyclic graph showing the model assumptions, with minimum covariate adjustment.
White nodes represent the minimum sufficient set of covariates that needs to be adjusted to block all confounding paths. Light grey nodes are variables that are assumed to directionally influence other nodes, but which were set as unmeasured (latent) in the present study and were therefore not included as candidate covariates for adjustment by the DAGitty algorithm. Green arrows represent assumed causal relationships between air pollutant exposure and cognitive performance. Black arrows represent putatively-blocked confounding paths if minimum covariate adjustment were applied.
Met = meteorological.

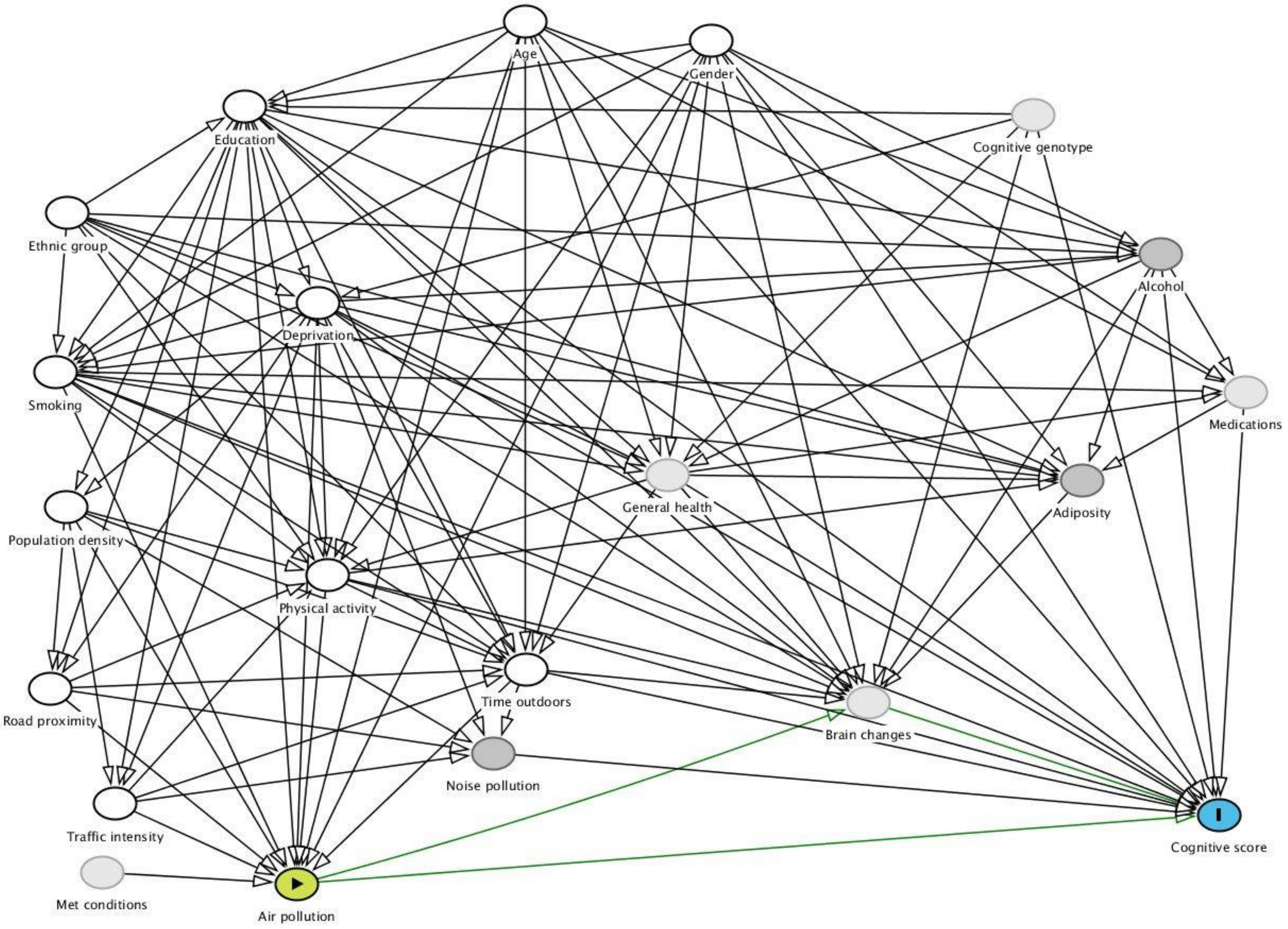

Figure S1c Directed acyclic graph showing the model assumptions, with covariate adjustment as applied in the main analysis models.
White nodes represent the set of covariates that was adjusted in the main analysis models in the present study. Light grey nodes are variables that are assumed to directionally influence other nodes, but which were set as unmeasured (latent) in the present study and were therefore not included as candidate covariates for adjustment by the DAGitty algorithm. Green arrows represent assumed causal relationships between air pollutant exposure and cognitive performance. Black arrows represent putatively-blocked confounding paths after covariate adjustment has been applied in the main analysis models.
Met = meteorological.

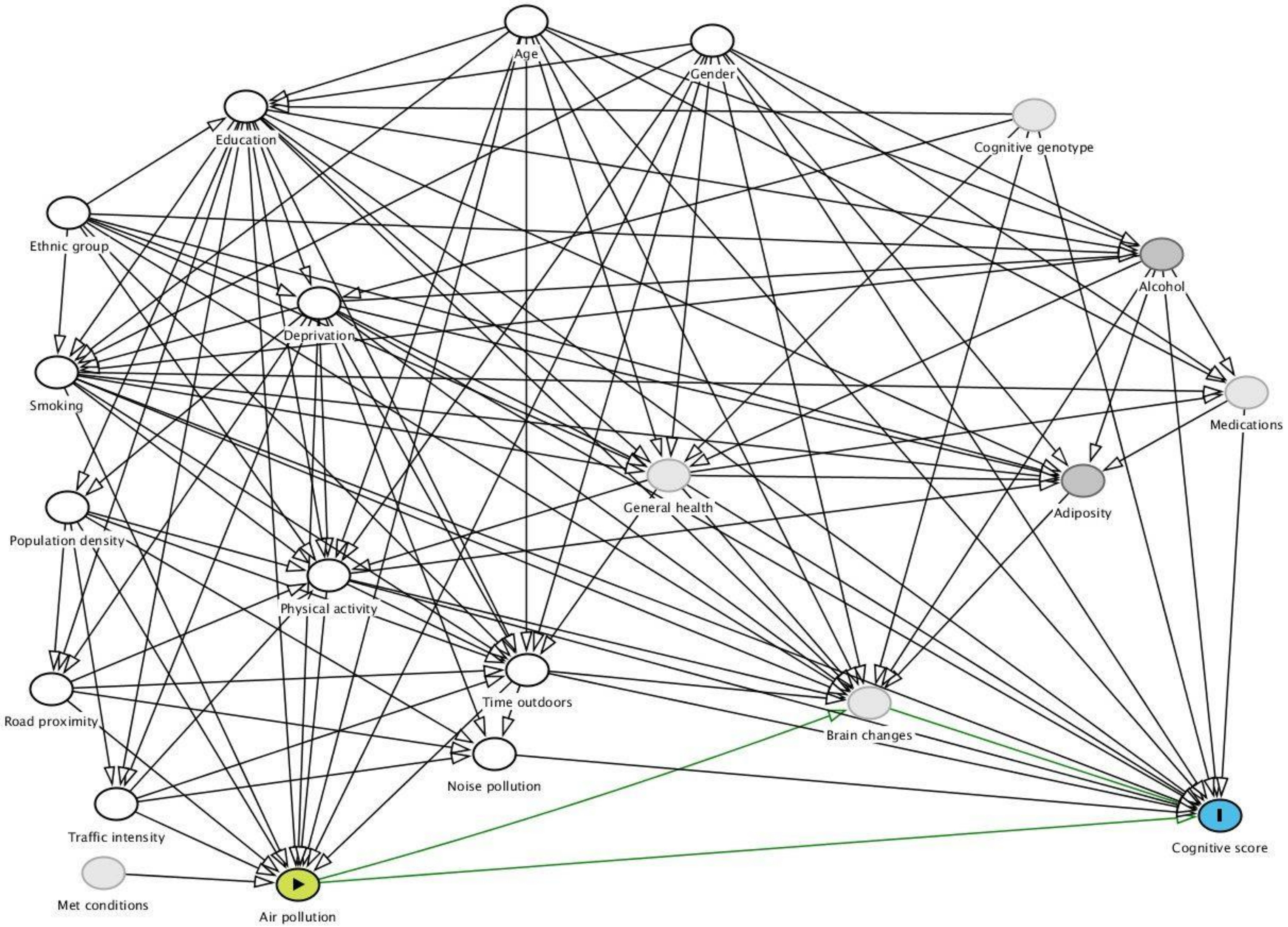

Figure S1d Directed acyclic graph showing the model assumptions, with covariate adjustment as applied in the main analysis models plus additional adjustment for noise pollution. White nodes represent the set of covariates that was adjusted in a supplementary analysis in the present study (for the additional influence of noise pollution). Light grey nodes are variables that are assumed to directionally influence other nodes, but which were set as unmeasured (latent) in the present study and were therefore not included as candidate covariates for adjustment by the DAGitty algorithm. Green arrows represent assumed causal relationships between air pollutant exposure and cognitive performance. Black arrows represent putatively-blocked confounding paths after covariate adjustment has been applied.
Met = meteorological.

List of conditions affecting brain function (self-reported at baseline)

UK Biobank field	UK Biobank code	Description
6150 vascular	3	stroke
20001 cancer	1031	meningeal cancer/malignant meningioma
"	1032	brain cancer/primary malignant tumour
20002 non-cancer	1081	stroke
"	1082	transient ischaemic attack
"	1083	subdural haemorrhage/haematoma
"	1086	subarachnoid haemorrhage
"	1240	neurological injury/trauma
"	1244	infection of nervous system
"	1245	brain/intracranial abscess
"	1246	encephalitis
"	1247	meningitis
"	1258	chronic/degenerative neurological problem
"	1259	motor neurone disease
"	1261	multiple sclerosis
"	1262	Parkinson's disease
"	1263	dementia/Alzheimer's/cognitive impairment
"	1264	epilepsy
"	1266	head injury
"	1397	other demyelinating condition
"	1425	cerebral aneurysm
"	1433	cerebral palsy
"	1434	other neurological problem
"	1491	brain haemorrhage
"	1524	spina bifida
"	1583	ischaemic stroke
"	1626	fracture skull/head
"	1659	meningioma benign
"	1683	neuroma benign

Supplementary Tables

Table S1a Results of regression models for cognitive performance in UK Biobank participants at baseline (2010), including adjustment for noise pollution

**Exposure (year)**	**Cognitive score**	**Adjusted (including noise pollution)[a]**					
		n	**Estimate**	**95% CI**	***p* (uncorr)**	***p* (FDR)[b]**	**+/-**
PM_{10} (2007)	Reasoning[c]	72,004	.0108	.0050, .0165	.0002	.0050	+
	Reaction time[d]	73,581	.9995[e]	.9986, 1.0004	.2940	.4083	+
	Numeric memory[f]	1,258	-.0289	-.0891, .0312	.3457	.4549	-
	Pairs matching[g]	72,338	1.0021	.9998, 1.0043	.0725	.1394	-
	Prospective memory[h]	73,974	1.0068	.9995, 1.0142	.0695	.1394	+
$PM_{2.5\ to\ 10}$ (2010)	Reasoning[c]	72,079	-.0085	-.0321, .0151	.4798	.5712	-
	Reaction time[d]	73,656	1.0017	.9995, 1.0040	.1342	.2237	-
	Numeric memory[f]	1,260	-.2096[i]	-.3764, -.0428	.0138	.0481	-
	Pairs matching[g]	72,413	.9957	.9863, 1.0052	.3762	.4703	+
	Prospective memory[h]	74,049	1.0009	.9713, 1.0314	.9550	.9550	+
$PM_{2.5}$ (2010)	Reasoning[c]	72,079	-.0144	-.0352, .0063	.1719	.2686	-
	Reaction time[d]	73,656	1.0032	1.0011, 1.0052	.0023	.0191	-
	Numeric memory[f]	1,260	-.0975	-.1905, -.0046	.0397	.0993	-
	Pairs matching[g]	72,413	1.0023	.9939, 1.0108	.5944	.6461	-
	Prospective memory[h]	74,049	.9912	.9649, 1.0183	.5216	.5927	-
NO_2 (2005)	Reasoning[c]	72,106	.0030	.0012, .0049	.0013	.0163	+
	Reaction time[d]	73,683	1.0000[j]	.9997, 1.0003	.9428	.9550	NA
	Numeric memory[f]	1,260	-.0113	-.0306, .0080	.2515	.3699	-
	Pairs matching[g]	72,440	1.0011	1.0003, 1.0018	.0042	.0263	-
	Prospective memory[h]	74,076	1.0023	1.0000, 1.0046	.0499	.1134	+
NO_x (2010)	Reasoning[c]	72,106	-.0015	-.0028, -.0003	.0154	.0481	-
	Reaction time[d]	73,683	1.0002	1.0001, 1.0003	.0055	.0275	-
	Numeric memory[f]	1,260	-.0079	-.0141, -.0016	.0142	.0481	-
	Pairs matching[g]	72,440	1.0006	1.0001, 1.0011	.0238	.0661	-
	Prospective memory[h]	74,076	.9986	.9971, 1.0002	.0889	.1588	-

+/-, point estimate indicates that higher values of the pollutant are associated with better (+) or worse (-) cognitive performance; CI, confidence interval; FDR, false discovery rate; NA, not applicable; NO_2, nitrogen dioxide; NO_x, nitrogen oxides; PM, particulate matter.

All estimates are per unit (µg/m^3) difference in the air pollutant.

a. Adjusted for baseline age, gender, ethnic group, Townsend deprivation score, education, smoking status, physical activity, time outdoors, proximity to nearest major road, traffic intensity on nearest major road, population density category, and noise pollution. Adjusted results are reported from models without an interaction term between air pollutant and time outdoors, unless otherwise noted in the table.

b. Probability adjusted using the Simes-Benjamini-Hochberg method implemented in the Stata qqvalue package.

c. Linear regression; estimates reported as unstandardized coefficients; possible score range 0 to 13; lower is worse.

d. Linear regression using log-transformed values; exponentiated estimates reported as rate ratios; values above 1 indicate relatively longer reaction time.

e. Interaction between PM_{10} and time outdoors: estimates stratified by quintile of time outdoors ranged between .9988 (.9973, 1.0003) in quintile 4 and 1.0012 (.9997, 1.0026) in quintile 5.

f. Linear regression; estimates reported as unstandardized coefficients; possible score range 2 to 12; lower is worse.

g. Negative binomial regression; estimates reported as rate ratios; values above 1 indicate relatively more errors.

h. Logistic regression; estimates reported as odds ratios; values below 1 indicate relatively lower odds of a correct response.

i. Interaction between $PM_{2.5\ to\ 10}$ and time outdoors: estimates stratified by quintile of time outdoors ranged between -.1360 (-.4853, .2133) in quintile 2 and .1327 (-.1910, .4565) in quintile 5.

j. Interaction between NO_2 and time outdoors: estimates stratified by quintile of time outdoors ranged between .9998 (.9993, 1.0003) in quintile 4 and 1.0005 (1.0001, 1.0010) in quintile 5.

Table S1b Results of regression models for change in cognitive scores in UK Biobank participants between 2010 and 2012-2013, including adjustment for noise pollution

Exposure (year)	Cognitive score change	Adjusted (including noise pollution)[a]					
		n	Estimate	95% CI	*p* (uncorr)	*p* (FDR)[b]	+/-
PM_{10} (2007)	Reasoning[c]	2,593	.0171	-.0221, .0563	.3918	.6423	+
	Reaction time[d]	2,605	-3.0810	-5.4360, -.7260	.0103	.1360	+
	Pairs matching[e]	2,591	.0332	-.0578, .1243	.4744	.6777	-
	Prospective memory[f]	2,597	1.0623	.9303, 1.2130	.3719	.6423	-
$PM_{2.5\ to\ 10}$ (2010)	Reasoning[c]	2,592	-.1136	-.2331, .0059	.0624	.4160	-
	Reaction time[d]	2,604	3.5220	-3.798, 10.0078	.3780	.6423	-
	Pairs matching[e]	2,590	-.1170	-.3997, .1658	.4175	.6423	+
	Prospective memory[f]	2,596	1.1224	.7517, 1.6759	.5723	.7631	-
$PM_{2.5}$ (2010)	Reasoning[c]	2,592	-.0002	-.0877, .0872	.9957	.9957	-
	Reaction time[d]	2,604	-6.2132	-11.1495, -1.2769	.0136	.1360	+
	Pairs matching[e]	2,590	-.0416	-.2475, .1642	.6920	.8141	+
	Prospective memory[f]	2,596	1.0046	.7596, 1.3286	.9745	.9957	-
NO_2 (2005)	Reasoning[c]	2,593	.0114	-.0037, .0265	.1397	.5469	+
	Reaction time[d]	2,605	-.5344	-1.403, .3344	.2280	.5469	+
	Pairs matching[e]	2,591	-.0232	-.0602, .0139	.2208	.5469	+
	Prospective memory[f]	2,597	1.0276	.9814, 1.0761	.2461	.5469	-
NO_x (2010)	Reasoning[c]	2,593	.0015	-.0058, .0087	.6882	.8141	+
	Reaction time[d]	2,605	-.3058	-.7288, .1172	.1565	.5469	+
	Pairs matching[e]	2,591	-.0026	-.0196, .0144	.7668	.8520	+
	Prospective memory[f]	2,597	1.0119	.9937, 1.0304	.2016	.5469	-

+/-, point estimate indicates that higher values of the pollutant are associated with improvement (+) or decline (-) in cognitive performance; CI, confidence interval; FDR; false discovery rate; NO_2, nitrogen dioxide; NO_x, nitrogen oxides; PM, particulate matter.
All estimates are per unit (µg/m^3) difference in the air pollutant.
a. Adjusted for baseline age, gender, ethnic group, Townsend deprivation score, education, smoking status, physical activity, time outdoors, proximity to nearest major road, traffic intensity on nearest major road, population density category, time between baseline and follow-up, and noise pollution. Adjusted results are reported from models without an interaction term between air pollutant and time outdoors; likelihood ratio test results indicated that the interaction term did not improve model fit.
b. Probability adjusted using the Simes-Benjamini-Hochberg method implemented in the Stata qqvalue package.
c. Linear regression; estimates reported as unstandardized coefficients; negative change score values indicate worse performance at follow-up.
d. Linear regression; estimates reported as unstandardized coefficients; positive change score values indicate slower performance at follow-up.
e. Linear regression; estimates reported as unstandardized coefficients; positive change score values indicate more errors at follow-up.
f. Logistic regression; estimates reported as odds ratios; values above 1 indicate relatively higher odds of performance decline at follow-up.

Table S2a Results of regression models for cognitive performance in UK Biobank participants at baseline (2010), excluding participants with conditions affecting brain function

Exposure (year)	Cognitive score	Unadjusted						Adjusted[a]					
		n	Estimate	95% CI	*p* (uncorr)	*p* (FDR)[b]	+/-	*n*	Estimate	95% CI	*p* (uncorr)	*p* (FDR)[b]	+/-
PM_{10} (2007)	Reasoning[c]	79,844	-.0151	-.0200, -.0102	1.149e-09	1.596e-09	-	69,241	.0102	.0042, .0161	.0008	.0067	+
	Reaction time[d]	82,088	1.0020	1.0015, 1.0024	5.341e-19	1.027e-18	-	70,718	.9995[e]	.9986, 1.0005	.3311	.4357	+
	Numeric memory[f]	1,405	-.0264	-.0732, .0204	.2686	.2798	-	1,220	-.0214	-.0824, .0397	.4923	.5594	-
	Pairs matching[g]	80,373	1.0083	1.0065, 1.0101	5.651e-20	1.177e-19	-	69,558	1.0017	.9994, 1.0040	.1417	.2700	-
	Prospective memory[h]	82,597	.9631	.9583, .9678	4.168e-50	2.084e-49	-	71,085	1.0061	.9986, 1.0136	.1095	.2281	+
$PM_{2.5\ to\ 10}$ (2010)	Reasoning[c]	79,940	-.0949	-.1127, -.0771	1.509e-25	4.716e-25	-	69,319	-.0135	-.0373, .0103	.2671	.3968	-
	Reaction time[d]	82,185	1.0047	1.0030, 1.0063	2.445e-08	3.217e-08	-	70,796	1.0016	.9994, 1.0039	.1620	.2700	-
	Numeric memory[f]	1,407	-.0501	-.1209, .0208	.1664	.1891	-	1,222	-.1894[i]	-.3531, -.0256	.0234	.0650	-
	Pairs matching[g]	80,470	1.0154	1.0087, 1.0221	5.583e-06	6.979e-06	-	69,636	.9957	.9863, 1.0051	.3654	.4568	+
	Prospective memory[h]	82,694	.9207	.9037, .9381	4.193e-18	7.487e-18	-	71,163	.9983	.9694, 1.0281	.9119	.9119	-
$PM_{2.5}$ (2010)	Reasoning[c]	79,940	-.1235	-.1401, -.1069	5.652e-48	2.355e-47	-	69,319	-.0195	-.0409, .0019	.0748	.1702	-
	Reaction time[d]	82,185	1.0079	1.0063, 1.0094	2.058e-24	5.717e-24	-	70,796	1.0034	1.0014, 1.0054	.0008	.0067	-
	Numeric memory[f]	1,407	-.0400	-.1056, .0255	.2316	.2517	-	1,222	-.0853	-.1791, .0086	.0749	.1702	-
	Pairs matching[g]	80,470	1.0211	1.0149, 1.0274	3.004e-11	4.418e-11	-	69,636	1.0026	.9940, 1.0112	.5583	.6068	-
	Prospective memory[h]	82,694	.8697	.8547, .8851	2.592e-55	2.160e-54	-	71,163	.9851	.9591, 1.0118	.2698	.3968	-
NO_2 (2005)	Reasoning[c]	79,967	-.0059	-.0074, -.0045	9.553e-16	1.493e-15	-	69,341	.0028	.0009, .0047	.0034	.0170	+
	Reaction time[d]	82,212	1.0007	1.0006, 1.0009	1.100e-30	3.929e-30	-	70,818	1.0000[j]	.9998, 1.0003	.7787	.8111	NA
	Numeric memory[f]	1,407	-.0062	-.0198, .0073	.3684	.3684	-	1,222	-.0104	-.0301, .0093	.3029	.4207	-
	Pairs matching[g]	80,497	1.0027	1.0022, 1.0032	6.808e-24	1.702e-23	-	69,658	1.0010	1.0003, 1.0018	.0066	.0275	-
	Prospective memory[h]	82,721	.9879	.9864, .9894	5.154e-57	6.442e-56	-	71,185	1.0017	.9993, 1.0040	.1600	.2700	+
NO_x (2010)	Reasoning[c]	79,967	-.0079	-.0089, -.0068	1.043e-51	6.519e-51	-	69,341	-.0009[k]	-.0030, .0012	.3869	.4606	-
	Reaction time[d]	82,212	1.0005	1.0004, 1.0006	6.764e-22	1.537e-21	-	70,818	1.0002	1.0001, 1.0003	.0033	.0170	-
	Numeric memory[f]	1,407	-.0046	-.0097, .0006	.0840	.1000	-	1,222	-.0183[l]	-.0290, -.0076	.0008	.0067	-
	Pairs matching[g]	80,497	1.0015	1.0012, 1.0019	3.343e-16	5.572e-16	-	69,658	1.0011[m]	1.0002, 1.0020	.0148	.0529	-
	Prospective memory[h]	82,721	.9915	.9905, .9925	1.759e-60	4.398e-59	-	71,185	.9982	.9966, .9997	.0214	.0650	-

+/-, point estimate indicates that higher values of the pollutant are associated with better (+) or worse (-) cognitive performance; CI, confidence interval; FDR, false discovery rate; NA, not applicable; NO_2, nitrogen dioxide; NO_x, nitrogen oxides; PM, particulate matter.

All estimates are per unit (μg/m^3) difference in the air pollutant.

a. Adjusted for baseline age, gender, ethnic group, Townsend deprivation score, education, smoking status, physical activity, time outdoors, proximity to nearest major road, traffic intensity on nearest major road, and population density category. Adjusted results are reported from models without an interaction term between air pollutant and time outdoors, unless otherwise noted in the table.

b. Probability adjusted using the Simes-Benjamini-Hochberg method implemented in the Stata qqvalue package.

c. Linear regression; estimates reported as unstandardized coefficients; possible score range 0 to 13; lower is worse.

d. Linear regression using log-transformed values; exponentiated estimates reported as rate ratios; values above 1 indicate relatively longer reaction time.

e. Interaction between PM_{10} and time outdoors: estimates stratified by quintile of time outdoors ranged between .9989 (.9974, 1.0004) in quintile 4 and 1.0013 (.9998, 1.0028) in quintile 5.
f. Linear regression; estimates reported as unstandardized coefficients; possible score range 2 to 12; lower is worse.
g. Negative binomial regression; estimates reported as rate ratios; values above 1 indicate relatively more errors.
h. Logistic regression; estimates reported as odds ratios; values below 1 indicate relatively lower odds of a correct response.
i. Interaction between $PM_{2.5\text{ to }10}$ and time outdoors: estimates stratified by quintile of time outdoors ranged between -.1492 (-.3901, .0917) in quintile 3 and .1269 (-.2122, .4659) in quintile 5.
j. Interaction between NO_2 and time outdoors: estimates stratified by quintile of time outdoors ranged between .9999 (.9994, 1.0004) in quintile 4 and 1.0006 (1.0001, 1.0010) in quintile 5.
k. Interaction between NO_x and time outdoors: estimates stratified by quintile of time outdoors ranged between -.0030 (-.0060, .0001) in quintile 5 and .0000 (-.0031, .0030) in quintile 2.
l. Interaction between NO_x and time outdoors: estimates stratified by quintile of time outdoors ranged between -.0193 (-.0324, -.0063) in quintile 3 and -.0010 (-.0232, .0212) in quintile 2.
m. Interaction between NO_x and time outdoors: estimates stratified by quintile of time outdoors ranged between .9999 (.9990, 1.0009) in quintile 1 and 1.0013 (.9999, 1.0027) in quintile 4.

Table S2b Results of regression models for change in cognitive scores in UK Biobank participants between 2010 and 2012-2013, excluding participants with conditions affecting brain function at baseline

Exposure (year)	Cognitive score change	Unadjusted						Adjusted[a]					
		n	Estimate	95% CI	*p* (uncorr)	*p* (FDR)[b]	+/-	*n*	Estimate	95% CI	*p* (uncorr)	*p* (FDR)[b]	+/-
PM_{10} (2007)	Reasoning[c]	2,761	.0057	-.0229, .0343	.6949	.8175	+	2,489	.0146	-.0253, .0546	.4730	.7277	+
	Reaction time[d]	2,774	-1.4701	-3.1048, .1646	.0780	.4285	+	2,499	-3.1561	-5.5248, -.7874	.0090	.1800	+
	Pairs matching[e]	2,757	.0579	-.0034, .1192	.0642	.4285	-	2,486	.0432	-.0500, .1363	.3638	.7130	-
	Prospective memory[f]	2,787	1.0626	.9775, 1.1552	.1539	.6071	-	2,490	1.0570	.9217, 1.2121	.4278	.7130	-
$PM_{2.5\ to\ 10}$ (2010)	Reasoning[c]	2,760	-.0320	-.1020, .0381	.3714	.6753	-	2,488	-.0937	-.2073, .0198	.1057	.5837	-
	Reaction time[d]	2,773	-1.4213	-5.3115, 2.4690	.4740	.7292	+	2,498	2.9247	-3.8725, 9.7218	.3990	.7130	-
	Pairs matching[e]	2,756	.0795	-.0632, .2222	.2750	.6753	-	2,485	-.0409	-.3073, .2254	.7633	.9429	+
	Prospective memory[f]	2,786	1.0105	.8134, 1.2554	.9250	.9737	-	2,489	.9883	.6959, 1.4036	.9475	.9475	+
$PM_{2.5}$ (2010)	Reasoning[c]	2,760	-.0141	-.0806, .0523	.6765	.8175	-	2,488	-.0116	-.1022, .0790	.8015	.9429	-
	Reaction time[d]	2,773	-2.4175	-6.2177, 1.3827	.2125	.6071	+	2,498	-5.8758	-10.9068, -.8447	.0221	.2210	+
	Pairs matching[e]	2,756	.0697	-.0730, .2124	.3382	.6753	-	2,485	-.0356	-.2431, .1718	.7364	.9429	+
	Prospective memory[f]	2,786	1.0830	.9152, 1.2815	.3533	.6753	-	2,489	1.0182	.7686, 1.3488	.9000	.9475	-
NO_2 (2005)	Reasoning[c]	2,761	.0036	-.0061, .0133	.4612	.7292	+	2,489	.0118	-.0033, .0269	.1248	.5837	+
	Reaction time[d]	2,774	-.0644	-.6268, .4979	.8223	.9137	+	2,499	-.5070	-1.3846, .3707	.2576	.6193	+
	Pairs matching[e]	2,757	.0070	-.0146, .0286	.5234	.7477	-	2,486	-.0240	-.0609, .0130	.2043	.5837	+
	Prospective memory[f]	2,787	1.0242	.9973, 1.0518	.0785	.4285	-	2,490	1.0257	.9797, 1.0739	.2787	.6193	-
NO_x (2010)	Reasoning[c]	2,761	.0000	-.0054, .0053	.9883	.9883	NA	2,489	.0015	-.0057, .0086	.6879	.9429	+
	Reaction time[d]	2,774	-.0864	-.3914, .2186	.5788	.7717	+	2,499	-.2969	-.7208, .1270	.1698	.5837	+
	Pairs matching[e]	2,757	.0077	-.0038, .0191	.1895	.6071	-	2,486	.0006	-.0161, .0174	.9404	.9475	-
	Prospective memory[f]	2,787	1.0103	.9986, 1.0221	.0857	.4285	-	2,490	1.0113	.9942, 1.0286	.1982	.5837	-

+/-, point estimate indicates that higher values of the pollutant are associated with improvement (+) or decline (-) in cognitive performance; CI, confidence interval; FDR, false discovery rate; NA, not applicable; NO_2, nitrogen dioxide; NO_x, nitrogen oxides; PM, particulate matter.

All estimates are per unit ($\mu g/m^3$) difference in the air pollutant.

a. Adjusted for baseline age, gender, ethnic group, Townsend deprivation score, education, smoking status, physical activity, time outdoors, proximity to nearest major road, traffic intensity on nearest major road, population density category, and time between baseline and follow-up. Adjusted results are reported from models without an interaction term between air pollutant and time outdoors; likelihood ratio test results indicated that the interaction term did not improve model fit.

b. Probability adjusted using the Simes-Benjamini-Hochberg method implemented in the Stata qqvalue package.

c. Linear regression; estimates reported as unstandardized coefficients; negative change score values indicate worse performance at follow-up.

d. Linear regression; estimates reported as unstandardized coefficients; positive change score values indicate slower performance at follow-up.

e. Linear regression; estimates reported as unstandardized coefficients; positive change score values indicate more errors at follow-up.

f. Logistic regression; estimates reported as odds ratios; values above 1 indicate relatively higher odds of performance decline at follow-up.

Table S3a Comparison of unadjusted regression models for cognitive performance in UK Biobank participants at baseline (2010), using all available participants versus only participants with complete covariate data

Exposure (year)	Cognitive score	Unadjusted (all available)						Unadjusted (complete covariate data)					
		n	Estimate	95% CI	*p* (uncorr)	*p* (FDR)[a]	+/-	*n*	Estimate	95% CI	*p* (uncorr)	*p* (FDR)[a]	+/-
PM_{10} (2007)	Reasoning[b]	83,238	-.0139	-.0187, -.0091	1.558e-08	2.050e-08	-	72,004	-.0085	-.0136, -.0034	.0012	.0015	-
	Reaction time[c]	85,651	1.0019	1.0015, 1.0023	3.245e-18	5.795e-18	-	73,581	1.0015	1.0011, 1.0020	1.657e-11	2.762e-11	-
	Numeric memory[d]	1,456	-.0301	-.0762, .0160	.2003	.2177	-	1,258	-.0191	-.0686, .0304	.4504	.4692	-
	Pairs matching[e]	83,806	1.0083	1.0065, 1.0101	2.295e-20	4.781e-20	-	72,338	1.0069	1.0050, 1.0087	1.148e-12	2.208e-12	-
	Prospective memory[f]	86,198	.9649	.9601, .9696	2.986e-46	1.493e-45	-	73,975	.9687	.9636, .9739	1.934e-31	9.670e-31	-
$PM_{2.5\ to\ 10}$ (2010)	Reasoning[b]	83,331	-.0915	-.1092, -.0737	4.334e-24	1.083e-23	-	72,079	-.0827	-.1016, -.0638	8.837e-18	2.762e-17	-
	Reaction time[c]	85,746	1.0049	1.0033, 1.0065	1.996e-09	2.772e-09	-	73,656	1.0046	1.0029, 1.0064	1.690e-07	2.485e-07	-
	Numeric memory[d]	1,458	-.0533	-.1237, .0171	.1376	.1564	-	1,260	-.0338	-.1079, .0403	.3712	.4218	-
	Pairs matching[e]	83,901	1.0159	1.0094, 1.0225	1.644e-06	2.055e-06	-	72,413	1.0133	1.0063, 1.0204	.0002	.0003	-
	Prospective memory[f]	86,293	.9217	.9053, .9384	4.855e-19	9.337e-19	-	74,050	.9319	.9135, .9507	4.220e-12	7.536e-12	-
$PM_{2.5}$ (2010)	Reasoning[b]	83,331	-.1196	-.1361, -.1031	1.212e-45	5.050e-45	-	72,079	-.0971	-.1147, -.0795	3.161e-27	1.317e-26	-
	Reaction time[c]	85,746	1.0079	1.0064, 1.0093	4.543e-26	1.420e-25	-	73,656	1.0066	1.0050, 1.0083	3.153e-15	7.882e-15	-
	Numeric memory[d]	1,458	-.0382	-.1018, .0255	.2396	.2496	-	1,260	-.0298	-.0984, .0389	.3953	.4297	-
	Pairs matching[e]	83,901	1.0211	1.0149, 1.0274	2.653e-11	3.901e-11	-	72,413	1.0187	1.0121, 1.0254	3.097e-08	4.839e-08	-
	Prospective memory[f]	86,293	.8741	.8592, .8893	1.032e-52	8.600e-52	-	74,050	.8875	.8707, .9046	1.440e-34	1.200e-33	-
NO_2 (2005)	Reasoning[b]	83,364	-.0056	-.0070, -.0041	1.388e-14	2.169e-14	-	72,106	-.0036	-.0051, -.0021	3.627e-06	5.038e-06	-
	Reaction time[c]	85,779	1.0007	1.0006, 1.0008	1.833e-29	6.546e-29	-	73,683	1.0006	1.0005, 1.0007	3.100e-18	1.107e-17	-
	Numeric memory[d]	1,458	-.0060	-.0192, .0072	.3718	.3718	-	1,260	-.0031	-.0175, .0114	.6782	.6782	-
	Pairs matching[e]	83,934	1.0027	1.0022, 1.0032	2.417e-25	6.714e-25	-	72,440	1.0023	1.0017, 1.0029	4.233e-16	1.176e-15	-
	Prospective memory[f]	86,326	.9887	.9872, .9901	2.074e-53	2.592e-52	-	74,077	.9898	.9882, .9914	1.526e-35	1.907e-34	-
NO_x (2010)	Reasoning[b]	83,364	-.0076	-.0086, -.0066	1.895e-49	1.184e-48	-	72,106	-.0066	-.0077, -.0055	3.654e-33	2.284e-32	-
	Reaction time[c]	85,779	1.0005	1.0004, 1.0006	7.818e-23	1.777e-22	-	73,683	1.0004	1.0003, 1.0005	3.817e-14	8.675e-14	-
	Numeric memory[d]	1,458	-.0042	-.0092, .0007	.0944	.1124	-	1,260	-.0039	-.0093, .0015	.1611	.1918	-
	Pairs matching[e]	83,934	1.0016	1.0012, 1.0019	2.149e-17	3.582e-17	-	72,440	1.0014	1.0010, 1.0018	1.015e-12	2.115e-12	-
	Prospective memory[f]	86,326	.9919	.9909, .9929	3.304e-58	8.260e-57	-	74,077	.9924	.9913, .9935	1.279e-42	3.198e-41	-

+/-, point estimate indicates that higher values of the pollutant are associated with better (+) or worse (-) cognitive performance; CI, confidence interval; FDR, false discovery rate; NO_2, nitrogen dioxide; NO_x, nitrogen oxides; PM, particulate matter.

All estimates are per unit ($\mu g/m^3$) difference in the air pollutant.

a. Probability adjusted using the Simes-Benjamini-Hochberg method implemented in the Stata qqvalue package.

b. Linear regression; estimates reported as unstandardized coefficients; possible score range 0 to 13; lower is worse.

c. Linear regression using log-transformed values; exponentiated estimates reported as rate ratios; values above 1 indicate relatively longer reaction time.

d. Linear regression; estimates reported as unstandardized coefficients; possible score range 2 to 12; lower is worse.

e. Negative binomial regression; estimates reported as rate ratios; values above 1 indicate relatively more errors.

f. Logistic regression; estimates reported as odds ratios; values below 1 indicate relatively lower odds of a correct response.

Table S3b Comparison of unadjusted regression models for change in cognitive scores in UK Biobank participants between 2010 and 2012-2013, using all available participants versus only participants with complete covariate data

Exposure (year)	Cognitive score change	Unadjusted (all available)						Unadjusted (complete covariate data)					
		n	Estimate	95% CI	*p* (uncorr)	*p* (FDR)[a]	+/-	*n*	Estimate	95% CI	*p* (uncorr)	*p* (FDR)[a]	+/-
PM_{10} (2007)	Reasoning[b]	2,878	.0088	-.0193, .0369	.5410	.7213	+	2,593	.0027	-.0275, .0329	.8620	.8709	+
	Reaction time[c]	2,896	-1.6117	-3.2010, -.0224	.0469	.4450	+	2,605	-2.0529	-3.6876, -.4182	.0138	.2760	+
	Pairs matching[d]	2,876	.0557	-.0052, .1167	.0731	.4450	-	2,591	.0568	-.0084, .1220	.0877	.3603	-
	Prospective memory[e]	2,910	1.0615	.9819, 1.1475	.1335	.4450	-	2,617	1.0761	.9840, 1.1767	.1081	.3603	-
$PM_{2.5\ to\ 10}$ (2010)	Reasoning[b]	2,877	-.0372	-.1067, .0322	.2932	.6516	-	2,592	-.0376	-.1115, .0363	.3187	.6038	-
	Reaction time[c]	2,895	-1.8676	-5.7511, 2.0159	.3459	.6572	+	2,604	-1.4953	-5.5797, 2.5891	.4730	.6757	+
	Pairs matching[d]	2,875	.0632	-.0762, .2026	.3744	.6572	-	2,590	.0670	-.0810, .2149	.3749	.6038	-
	Prospective memory[e]	2,909	.9726	.7856, 1.2043	.7991	.9232	+	2,616	.9584	.7414, 1.2390	.7459	.8643	+
$PM_{2.5}$ (2010)	Reasoning[b]	2,877	-.0044	-.0691, .0602	.8928	.9232	-	2,592	-.0095	-.0757, .0566	.7779	.8643	-
	Reaction time[c]	2,895	-3.0284	-6.7852, .7284	.1141	.4450	+	2,604	-3.7737	-7.5464, -.0009	.0499	.3327	+
	Pairs matching[d]	2,875	.0790	-.0605, .2184	.2671	.6516	-	2,590	.0857	-.0616, .2329	.2543	.5651	-
	Prospective memory[e]	2,909	1.0841	.9232, 1.2731	.9232	.9232	-	2,616	1.0839	.9060, 1.2968	.3784	.6038	-
NO_2 (2005)	Reasoning[b]	2,878	.0041	-.0053, .0135	.3943	.6572	+	2,593	.0027	-.0074, .0128	.6012	.7515	+
	Reaction time[c]	2,896	-.1386	-.6848, .4075	.6189	.7736	+	2,605	-.2522	-.8303, .3259	.3925	.6038	+
	Pairs matching[d]	2,876	.0079	-.0133, .0291	.4662	.7172	-	2,591	.0067	-.0163, .0297	.5681	.7515	-
	Prospective memory[e]	2,910	1.0280	1.0023, 1.0545	.0328	.4450	-	2,617	1.0315	1.0021, 1.0617	.0357	.3327	-
NO_x (2010)	Reasoning[b]	2,878	.0003	-.0049, .0056	.8968	.9232	+	2,593	-.0005	-.0059, .0050	.8709	.8709	-
	Reaction time[c]	2,896	-.1005	-.3962, .1952	.5053	.7213	+	2,605	-.1785	-.4818, .1249	.2488	.5651	+
	Pairs matching[d]	2,876	.0078	-.0035, .0191	.1753	.5009	-	2,591	.0088	-.0034, .0209	.1579	.4511	-
	Prospective memory[e]	2,910	1.0094	.9982, 1.0207	.0997	.4450	-	2,617	1.0104	.9981, 1.0228	.0985	.3603	-

+/-, point estimate indicates that higher values of the pollutant are associated with improvement (+) or decline (-) in cognitive performance; CI, confidence interval; FDR; false discovery rate; NO_2, nitrogen dioxide; NO_x, nitrogen oxides; PM, particulate matter.

All estimates are per unit (µg/m^3) difference in the air pollutant.

a. Probability adjusted using the Simes-Benjamini-Hochberg method implemented in the Stata qqvalue package.

b. Linear regression; estimates reported as unstandardized coefficients; negative change score values indicate worse performance at follow-up.

c. Linear regression; estimates reported as unstandardized coefficients; positive change score values indicate slower performance at follow-up.

d. Linear regression; estimates reported as unstandardized coefficients; positive change score values indicate more errors at follow-up.

e. Logistic regression; estimates reported as odds ratios; values above 1 indicate relatively higher odds of performance decline at follow-up.

Table S4a Summary of Defra pollution data used in sensitivity analyses

	**Cross-sectional analysis sample**	**Follow-up analysis sample**
n	87,946	2,924
PM_{10} (2007) (μg/m^3)		
n (%) missing	1,465 (1.67)	13 (0.44)
Median (Q1, Q3)	19.08 (16.25, 21.88)	15.44 (14.46, 16.22)
$PM_{2.5\ to\ 10}$ (2010) (μg/m^3)[a]		
Median (Q1, Q3)	4.85 (4.48, 5.41)	4.49 (4.17, 4.94)
$PM_{2.5}$ (2010) (μg/m^3)[a]		
Median (Q1, Q3)	12.13 (11.07, 13.39)	10.62 (9.94, 11.17)
NO_2 (2005) (μg/m^3)		
n (%) missing	2,446 (2.78)	31 (1.06)
Median (Q1, Q3)	25.01 (20.74, 30.18)	20.28 (15.90, 23.37)
NO_x (2010) (μg/m^3)[a]		
Median (Q1, Q3)	38.52 (29.02, 49.57)	28.18 (21.18, 35.99)

NO_2, nitrogen dioxide; NO_x, nitrogen oxides; PM, particulate matter; Q, quartile.
a. No missing data

Table S4b Results of regression models for change in cognitive scores in UK Biobank participants between 2010 and 2012-2013, using Defra pollutant data

Exposure (year)	Cognitive score change	Unadjusted						Adjusted[a]					
		n	Estimate	95% CI	*p* (uncorr)	*p* (FDR)[b]	+/-	*n*	Estimate	95% CI	*p* (uncorr)	*p* (FDR)[b]	+/-
PM_{10} (2007)	Reasoning[c]	2,876	.0116	-.0260, .0492	.5453	.9748	+	2,581	.0346	-.0110, .0802	.1372	.7460	+
	Reaction time[d]	2,894	-.3273	-2.6852, 2.0304	.7855	.9748	+	2,593	-1.1702	-4.1426, 1.8022	.4403	.7460	+
	Pairs matching[e]	2,873	-.0096	-.0958, .0765	.8264	.9748	+	2,579	-.0364	-.1384, .0657	.4849	.7460	+
	Prospective memory[f]	2,908	.9934	.8922, 1.1062	.9043	.9748	+	2,586	.9393	.7932, 1.1124	.4682	.7460	+
$PM_{2.5\ to\ 10}$ (2010)	Reasoning[c]	2,889	.0299	-.0706, .1305	.5595	.9748	+	2,593	.0659	-.0467, .1784	.2512	.7460	+
	Reaction time[d]	2,907	3.3394	-2.3490, 9.0279	.2499	.9748	-	2,605	4.0715	-2.1275, 10.2705	.1980	.7460	-
	Pairs matching[e]	2,886	-.0444	-.2562, .1673	.6807	.9748	+	2,591	-.0331	-.2753, .2091	.7888	.8830	+
	Prospective memory[f]	2,921	1.0295	.7714, 1.3740	.8435	.9748	-	2,597	1.0794	.7218, 1.6141	.7097	.8830	-
$PM_{2.5}$ (2010)	Reasoning[c]	2,889	.0354	-.0282, .0990	.2751	.9748	+	2,593	.0900	.0040, .1760	.0404	.5210	+
	Reaction time[d]	2,907	1.2202	-2.6392, 5.0796	.5355	.9748	-	2,605	-.2549	-5.5869, 5.0772	.9254	.9254	+
	Pairs matching[e]	2,886	-.0108	-.1545, .1329	.8829	.9748	+	2,591	.0743	-.2633, .1146	.4407	.7460	-
	Prospective memory[f]	2,921	.9786	.8259, 1.1596	.8029	.9748	+	2,597	.9003	.6805, 1.1912	.4623	.7460	+
NO_2 (2005)	Reasoning[c]	2,858	-.0045	-.0156, .0067	.4304	.9748	-	2,565	.0008	-.0138, .0154	.9143	.9254	+
	Reaction time[d]	2,876	-.0329	-.7287, .6628	.9261	.9748	+	2,577	-.3816	-1.3004, .5373	.4157	.7460	+
	Pairs matching[e]	2,855	.0119	-.0115, .0353	.3199	.9748	-	2,563	.0043	-.0283, .0370	.7947	.8830	-
	Prospective memory[f]	2,890	.9952	.9633, 1.0281	.7706	.9748	+	2,570	.9632	.9097, 1.0198	.1984	.7460	+
NO_x (2010)	Reasoning[c]	2,889	.0026	-.0024, .0075	.3113	.9748	+	2,593	.0063	-.0001, .0127	.0521	.5210	+
	Reaction time[d]	2,907	-.0037	-.3065, .2990	.9807	.9807	+	2,605	-.1184	-.5192, .2824	.5627	.8039	+
	Pairs matching[e]	2,886	.0012	-.0099, .0123	.8313	.9748	-	2,591	-.0036	-.0178, .0106	.6204	.8272	+
	Prospective memory[f]	2,921	.9946	.9802, 1.0091	.4604	.9748	+	2,597	.9879	.9647, 1.0117	.3159	.7460	+

+/-, point estimate indicates that higher values of the pollutant are associated with improvement (+) or decline (-) in cognitive performance; CI, confidence interval; FDR; false discovery rate; NO_2, nitrogen dioxide; NO_x, nitrogen oxides; PM, particulate matter.

All estimates are per unit (μg/m^3) difference in the air pollutant.

a. Adjusted for baseline age, gender, ethnic group, Townsend deprivation score, education, smoking status, physical activity, time outdoors, proximity to nearest major road, traffic intensity on nearest major road, population density category, and time between baseline and follow-up. Adjusted results are reported from models without an interaction term between air pollutant and time outdoors; likelihood ratio test results indicated that the interaction term did not improve model fit.

b. Probability adjusted using the Simes-Benjamini-Hochberg method implemented in the Stata qqvalue package.

c. Linear regression; estimates reported as unstandardized coefficients; negative change score values indicate worse performance at follow-up.

d. Linear regression; estimates reported as unstandardized coefficients; positive change score values indicate slower performance at follow-up.

e. Linear regression; estimates reported as unstandardized coefficients; positive change score values indicate more errors at follow-up.

f. Logistic regression; estimates reported as odds ratios; values above 1 indicate relatively higher odds of performance decline at follow-up.

Supplementary References

1 von Elm, E. *et al.* The Strengthening the Reporting of Observational Studies in Epidemiology (STROBE) statement: guidelines for reporting observational studies. *Epidemiology* **18**, 800-804, doi:10.1097/EDE.0b013e3181577654 (2007).

2 Textor, J., Hardt, J. & Knuppel, S. DAGitty: a graphical tool for analyzing causal diagrams. *Epidemiology* **22**, 745, doi:10.1097/EDE.0b013e318225c2be (2011).

3 Pearl, J. *Causality: Models, Reasoning, and Inference*. 2nd edn, (Cambridge University Press, 2009).
